# Cardiorespiratory fitness is associated with cognitive function in late adulthood: baseline findings from the IGNITE study

**DOI:** 10.1136/bjsports-2024-108257

**Published:** 2024-12-10

**Authors:** Lauren E Oberlin, Lu Wan, Chaeryon Kang, Allison Romano, Sarah Aghjayan, Alina Lesnovskaya, Hayley S Ripperger, Jermon Drake, Rae Harrison, Audrey M Collins, Cristina Molina-Hidalgo, George Grove, Haiqing Huang, Arthur Kramer, Charles H Hillman, Jeffrey M Burns, Eric D Vidoni, Edward McAuley, M Ilyas Kamboh, John M Jakicic, Kirk I Erickson

**Affiliations:** 1 Department of Neuroscience, AdventHealth Orlando, Orlando, Florida, USA; 2 Department of Psychiatry, Weill Cornell Medicine, New York, New York, USA; 3 Department of Psychology, University of Pittsburgh, Pittsburgh, Pennsylvania, USA; 4 Department of Biostatistics, University of Pittsburgh, Pittsburgh, Pennsylvania, USA; 5 Department of Neuroscience, AdventHealth Research Institute, Orlando, Florida, USA; 6 Center for Cognitive and Brain Health, Northeastern University - Boston Campus, Boston, Massachusetts, USA; 7 Beckman Institute, University of Illinois at Urbana-Champaign, Urbana, Illinois, USA; 8 Department of Physical Therapy, Movement, and Rehabilitation Sciences, Northeastern University - Boston Campus, Boston, Massachusetts, USA; 9 Department of Psychology, Northeastern University, Boston Campus, Boston, Massachusetts, USA; 10 Alzheimer's Disease Research Center, The University of Kansas Medical Center, Kansas City, Kansas, USA; 11 Department of Health and Kinesiology, University of Illinois at Urbana-Champaign, Urbana, Illinois, USA; 12 Department of Human Genetics, University of Pittsburgh School of Public Health, Pittsburgh, Pennsylvania, USA; 13 Department of Psychiatry, University of Pittsburgh, Pittsburgh, Pennsylvania, USA; 14 Department of Internal Medicine, The University of Kansas Medical Center, Kansas City, Kansas, USA

**Keywords:** Aging, Physical fitness, Neurology, Preventive Medicine, Public health

## Abstract

**ABSTRACT:**

**Objectives:**

To evaluate the association between cardiorespiratory fitness (CRF) and cognition in a large sample of older adults, and to examine clinical and demographic factors that might moderate these associations.

**Methods:**

CRF was measured with a graded exercise test performed on a motorised treadmill. A confirmatory factor analysis was conducted using data from a comprehensive neuropsychological battery to obtain latent factors reflecting core cognitive domains. Linear regression models evaluated the association between CRF and each of the cognitive composites, and potential moderators including demographic factors (age, sex, education), apolipoprotein E ε4 (*APOE4*) carriage, beta-blocker use and components of maximal effort criteria during CRF testing.

**Results:**

The sample consisted of 648 adults (mean (SD) age 69.88 (3.75)), including 461 women (71.1%). The highest oxygen consumption obtained during testing (VO_2max_) was mean (SD) = 21.68 (5.06) mL/kg/min. We derived a five-factor model composed of episodic memory, processing speed, working memory, executive function/attentional control and visuospatial function. Higher CRF was associated with better performance across all five cognitive domains after controlling for covariates. Age and *APOE4* carriage did not moderate observed associations. The relationship between CRF and cognitive performance was greater in women, those with fewer years of education and those taking beta-blockers in the domains of processing speed (sex: β=−0.447; p=0.015; education: β=−0.863; p=0.018) and executive function/attentional control (sex: β=−0.417; p=0.022; education β=−0.759; p=0.034; beta-blocker use: β=0.305; p=0.047).

**Conclusion:**

Higher CRF in older adulthood is associated with better cognitive performance across multiple domains susceptible to age-related cognitive decline. Sex, education and use of beta-blockers moderated observed associations within select cognitive domains.

**WHAT IS ALREADY KNOWN ON THIS TOPIC**

- Studies suggest that cardiorespiratory fitness (CRF) relates to cognitive and brain health in older adulthood, although many questions remain about the specific cognitive processes associated with fitness, and how clinical, demographic and genetic factors might influence these relationships.

**WHAT THIS STUDY ADDS**

- Higher CRF in older adulthood relates to cognitive benefits across multiple cognitive domains susceptible to age-related decline. Higher CRF was positively associated with cognition even in the presence of risk factors for decline (greater age, *APOE4* carriage), while women, those with fewer years of education, and those taking beta-blockers showed greater cognitive benefits in select domains.

**HOW THIS STUDY MIGHT AFFECT RESEARCH, PRACTICE OR POLICY**

- These findings highlight CRF as an important health factor for preserving multidomain cognitive functioning in older adulthood. Understanding moderators of this relationship might help to inform the development of individualised exercise prescriptions that target CRF to optimise cognitive health in ageing.

## Introduction

 Preserving neurocognitive health in older adulthood is a global public health imperative. Cardiorespiratory fitness (CRF), a physiological measure of aerobic capacity that can be modified by regular aerobic exercise, has been linked to reduced risk of age-related cognitive decline and dementia.^1^,^4^ However, there is a lack of scientific consensus about the nature and specificity of these relationships. Heterogeneity in methods used to measure both CRF and cognition, along with limited power among existing studies,^5^,^14^ has hindered widespread understanding of the relationship between CRF and cognition in ageing. Furthermore, few studies have had sufficiently large sample sizes to allow for the decomposition of CRF based on factors (eg, beta-blockers) that influence criteria (eg, heart rate) for obtaining a peak or maximal effort during a graded exercise test.^15^ In addition, while a graded exercise test is the gold standard for measuring CRF, due to challenges with the resources, infrastructure and expertise required, the few large-scale studies examining these relationships have largely relied on proxy tests (eg, based on heart rate obtained via exercise ECG),^16^ maximal duration of symptom-limited exercise^17^ and surrogate measures (eg, physical function tests,^18^ formula-based estimates^19 20^) to estimate CRF. Thus, fundamental questions regarding the link between CRF, assessed by a graded exercise test, and cognition in ageing remain. A better understanding of these relationships might help to inform public health recommendations for maintaining fitness levels to preserve cognitive health in older adulthood.

In addition to heterogeneity in the assessment of CRF, there is significant study variability in the measurement of cognitive performance. Most prior research examining associations between CRF and cognition have used screening measures of global cognitive abilities,^1 18^ a single cognitive test to measure an individual domain (eg, memory, executive function)^9 11 12 19 21^ or a brief battery of tests^13 16 17 22^ that insufficiently capture the breadth and depth of cognitive function. For example, a study of 65 older adults examined associations between CRF and performance in the memory domain and found a positive relationship only among men.^11^ Two other studies, involving samples of 54^9^ and 60 older adults,^13^ found that higher CRF related to better task performance, but examined only executive function. Thus, it remains unknown whether higher CRF is broadly associated with cognition across numerous domains or if specific cognitive processes show the greatest benefit.

Finally, there has been speculation that several demographic and genetic factors might affect the relationship between CRF and cognitive function in older adulthood. However, data regarding the role of potential moderators are mixed,^3 11 16 23^ leading to an incomplete understanding of critical factors that might influence these relationships. For example, several studies suggest that the neurocognitive benefits of high CRF are more apparent in women and carriers of the apolipoprotein E ε4 (*APOE4*) allele, a genetic variant that increases the risk of Alzheimer’s disease.^23^,^25^ Yet, other studies failed to find these associations or have reported the opposite pattern.^1 11 16 17^ Hence, recent reports have cautioned that mixed and inconsistent findings prohibit definitive conclusions about the impact of select clinical and demographic factors on the relationship between fitness and cognition.^26^,^28^ Large-scale studies examining individual differences and the role of potential moderators could offer valuable insights into the personalisation of fitness parameters most likely to optimise cognitive health in older adulthood.

In a large sample of cognitively healthy older adults, we evaluated associations between a gold-standard measure of CRF and cognitive functioning, using latent factors reflecting core cognitive domains derived from a comprehensive neuropsychological battery. CRF was defined as the highest oxygen consumption obtained during a graded exercise test (VO_2max_). We further examined demographic, genetic (*APOE4* carriage) and methodological factors associated with the measurement of CRF (beta-blockers; criteria for achieving maximal exertion during the measurement of CRF) that might moderate these associations. We predict (1) widespread associations between VO_2max_ and cognitive performance across domains, and (2) effect moderation by demographic, methodological and genetic factors (ie, age, sex, *APOE4*).

## Methods

### Participants

Adults aged 65–80 years were recruited for participation in a 12- month multisite (Boston, Kansas City, Pittsburgh, USA) aerobic exercise intervention (Investigating Gains in Neurocognition in an Intervention Trial of Exercise (IGNITE)) (ClinicalTrials.gov: NCT02875301). Participants were excluded from the study if they had current major depression or substance use disorder; history or presence of neurological conditions (eg, Parkinson’s disease, stroke, dementia) or severe mental illness (eg, schizophrenia); self-reported engagement in >20 min per day of structured moderate-to-vigorous intensity physical activity, 3 days or more per week, over the past 6 months; recent history or treatment for severe cardiovascular events (eg, congestive heart failure, angioplasty); type 1 diabetes, uncontrolled or insulin-dependent type 2 diabetes; or MRI contraindications. Details of recruitment and eligibility criteria can be found in online supplemental file 1 and Erickson *et al*, 2019.^29^

A community-based sample of 648 participants were enrolled in the trial and included in this analysis of baseline data. The study was approved by the institutional review board at each site, and all participants provided written informed consent before data collection.

### Standardisation of the protocol

All administration and data collection procedures were standardised across sites. Each year, all staff were recertified for standardised administration of assessments. To further enhance standardisation, cognitive data were double-scored and all data were double-entered into a REDCap database by the coordinating centre.

### Patient and public involvement

This was a community-based sample without patient involvement.

### Equity, diversity and inclusion statement

Study data were obtained from a single high-income country (United States). Recruitment was based on the regional demographic representation of racial and ethnic minorities at each of the three study sites, according to US census data. Participants self-identified their race/ethnicity along the National Institutes of Health guidelines. Our research team consisted of 10 women and 11 men from varying nationalities, disciplines and fields of expertise, with representation from researchers at all career stages.

### Cardiorespiratory fitness testing

CRF was measured with a graded exercise test performed on a motorised treadmill. Treadmill speed was determined by participants walking at a pace of 1.5–3.5 mph that resulted in a heart rate of 70% of age-predicted maximal heart rate ±5 beats, or a rating of perceived exertion (RPE) of 11 on the Borg rating scale^30^ for anyone reporting use of beta-blocker medications. Participants showing American College of Sports medicine-defined contraindications for performing a graded exercise test were not permitted to continue the test.^31^

The test began with a 1 min standing rest phase and 2 min warm-up phase walking at 0.50 mph less than the agreed-upon test speed with zero incline. Using a modified Balke protocol,^32^ treadmill speed was maintained throughout the test but a 2% increase in grade was executed every 2 min. During each stage, blood pressure and RPE were obtained while ECG was monitored. Oxygen consumption was measured continuously throughout the test using indirect calorimetry with metabolic carts (Parvo Medics TrueOne 2400; COSMED Quark CPET). The test was completed to volitional exhaustion or with symptom limitation, and was followed by a 4 min active cool-down and a 4 min resting cool-down. The highest VO_2_ value obtained during the test was used as the indicator of VO_2max_ and represents the measure of CRF that we used here, regardless of whether the participant reached a plateau in VO_2_.^33^ We also recorded whether the test met standard American College of Sports Medicine criteria^31^ for defining maximal effort on a graded exercise test including: (1) plateau in VO_2_ between two or more workloads (increase <0.15 L/min or 2.0 mL/kg/min during the last minute of corresponding workloads); (2) respiratory exchange ratio (RER) ≥1.10; (3) heart rate within 10 beats of the age-predicted maximal heart rate (220−age), and (4) an RPE ≥17. Maximal effort is typically defined as achieving at least three out of these four criteria.^34^

### *APOE* genotyping

The *APOE* gene has three main alleles (ε2, ε3, ε4). Carriers of the ε4 allele have an increased risk of Alzheimer’s disease, with homozygous carriers (ε4/ε4) having the greatest elevation of risk.^35^ Genotypes for the two *APOE* single nucleotide polymorphisms, resulting in six genotypes, were detected and measured in blood samples using TaqMan assays.^36^ Participants with at least one ε4 allele (ε2/ε4, ε3/ε4, ε4/ε4) were classified as *APOE4* carriers.

### Cognitive assessment

Participants completed a comprehensive neuropsychological evaluation consisting of the Montreal Cognitive Assessment and measures of processing speed (Letter Comparison Test, Digit Symbol Substitution Test, Trail Making Test, Part A), working memory (N-back Working Memory task, Spatial Working Memory task, List Sorting Working Memory task), visuospatial processing (Matrix Reasoning, Spatial Relations), episodic memory (Brief Visuospatial Memory Test, Picture Sequencing Test, Hopkins Verbal Learning Test, Logical Memory Test, Verbal Paired Associates) and attentional control (Flanker task, Stroop task (incongruent trial), Dimensional Change Card Sort task, Trail Making Test, Part B). Cognitive testing was administered by annually certified psychometricians and completed across 2 days. All cognitive tests have established validity and reliability in older adult populations (see online supplemental methods and Erickson *et al*, 2019 for task descriptions).

### Statistical analyses

#### Dimensionality reduction of the cognitive data

A confirmatory factor analysis (CFA) tested the latent architecture of the cognitive tests. Prior to conducting the CFA, all cognitive outcomes were examined to identify missing data and out-of-range values. Twelve participants had missing data for several cognitive tests, and 20 participants had out-of-range values (scores±4 SD from the mean)^37^,^39^ in at least one outcome. We imputed the missing values in each of the test outcomes with the median value of the outcome and kept all other out-of-range values. There was no significant difference in goodness-of-fit indices when excluding out-of-range values. Z-standardisation was used to ensure that outcomes with different response scales were on a standardised and comparable metric.

The CFA was conducted using the *lavaan* package in R^40^ with maximum likelihood estimation. We tested the fit of several models that differed in the following ways: (a) whether model fit improved with a second-order general cognitive factor, (b) whether the model fit was better with a single factor of executive function (containing the tasks of attentional control and working memory) or by maintaining attentional control and working memory as distinct factors (see also online supplemental tables S6 and S7).

Seven different goodness-of-fit statistics were used to assess model fit using established cut-off points, including *χ*^2^, the *χ*^2^/df ratio,^41^ the Comparative Fit Index, the Tucker-Lewis Index,^42 43^ the root mean square error of approximation, the standardised root mean square residual^44 45^ and the Akaike information criterion^46^ (online supplemental methods). After the best-fit model was identified, the factor scores were extracted for further analysis.

#### Testing associations between CRF and cognitive components

Linear regression models were used to assess associations between age, education, and the CFA-derived cognitive factors. We tested our hypotheses using VO_2max_ as a predictor term in multiple regression models, with each of the five cognitive composites as dependent variables of interest. All variables were entered into regression models simultaneously, and age, sex, site, education, *APOE4* carriage and body mass index (BMI) were included as covariates because of their association with either VO_2max_ or cognition and to account for site-related variation. We considered significant associations with p<0.05.

We examined possible moderators of the association between CRF and cognitive performance by modelling several interaction terms with CRF: demographic factors (age, sex, formal years of education), *APOE4* carriage, use of beta-blocker medication and achievement of three of four criteria for maximal effort. We employed multiple linear regression models, including CRF (VO_2max_), the moderator variable and their interaction product, controlling for demographic factors, study site, BMI and *APOE4* carriage (if not included as an interaction term). We also decomposed VO_2max_ components to examine independent associations between physiological capacity (assessed via RER) and cognition, and whether associations between VO_2max_ and cognition persisted after adjustment for RER. To comprehensively assess this, RER was examined as both a continuous variable and a dichotomous variable based on whether a maximum RER ≥1.10 was reached (yes/no). Analyses were performed using R software (version 4.2.1). All analyses and reporting of results are consistent with the CHecklist for statistical Assessment of Medical Papers (CHAMP) statement, which provides guidelines for reviewing and reporting statistics in medical research.^47^

## Results

### Participant characteristics

This sample included 648 older adults (mean (SD) age=69.88 (3.75)) with 461 women (71.1%) and an average of 16.32 (SD 2.21) years of education. Participants had an average VO_2max_ of 21.68 mL/kg/min (SD=5.06) and 15.1% reported taking a beta-blocker (table 1). Six hundred and forty were genotyped and 174 (27.2%) were *APOE4* carriers. VO_2max_ significantly correlated with each of the component criteria (max RER r=0.213, p<0.001; max RPE r=0.199, p<0.001; heart rate r=0.382, p<0.001; VO_2_ plateau r=−0.193, p<0.001).

**Table 1:** Participant characteristics

Characteristic	Mean (SD)
Number of participants	648
Site	
University of Pittsburgh (%)	33.8
University of Kansas (%)	33.0
Northeastern University (%)	33.2
Age (years)	69.88 (3.75)
Sex (% female)	71.1
Race (% non-white)	24.2
Ethnicity (% Hispanic)	3.1
Education (years)	16.32 (2.21)
*APOE4* carriage (% carrier)*	27.2
Beta-blocker (% yes)	15.1
BMI (kg/m^2^)	29.74 (5.75)
Hypertension (% yes)	58.2
High cholesterol (% yes)	46.5
Diabetes (% yes)	15.9
VO_2max_ (mL/kg/min)	21.68 (5.06)
Criteria for maximal exertion for measurement of CRF (% reaching three/four criteria)	79.6
Plateau in VO_2_ (%)	89.8
Max RER ≥1.1 (%)	47.7
Max RPE ≥17 (%)	91.0
HR within 10 of APMHR (%)	79.5

* Eight participants did not have apolipoprotein E data.

APMHRage-predicted maximal heart rateBMIbody mass indexCRFcardiorespiratory fitnessRERrespiratory exchange ratioRPErating of perceived exertionVO_2max_highest oxygen consumption obtained during a graded exercise test

### Confirmatory factor analysis

**Figure 1:**
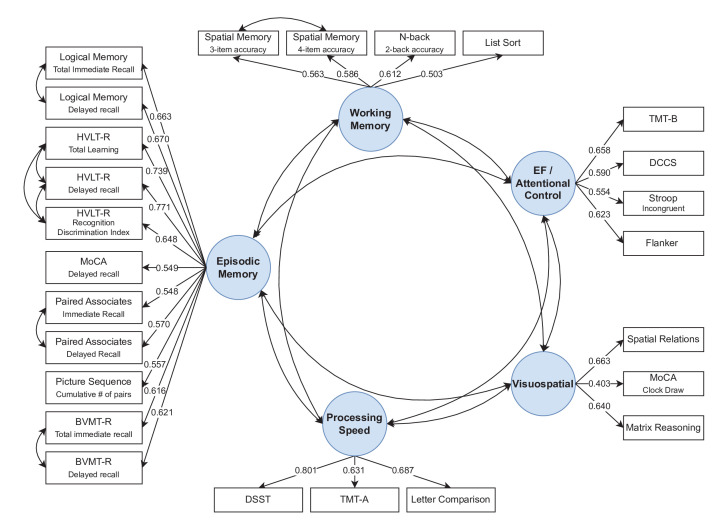
The best-fit cognitive factor model derived from the confirmatory factor analysis. Ovals depict latent factors, while rectangles reflect manifest variables representing cognitive test scores. Single-headed arrows represent paths of factor loadings, and double-headed arrows represent factor correlations or residual correlations. Standardised factor loadings are shown on the single-headed arrows. Error items were not included in the figure. BVMT, Brief Visuospatial Memory Test; DCCS, Dimensional Change Card Sort; DSST, Digit Symbol Substitution Test; EF, executive function; HVLT, Hopkins Verbal Learning Test; MoCA, Montreal Cognitive Assessment; TMT, Trail Making Test.

The best-fit model was composed of five factors: episodic memory, processing speed, working memory, executive function (EF)/attentional control, and a visuospatial factor (*χ*^2^=685.99, df=259, p<0.001, *χ*^2^/df=2.649, Comparative Fit Index=0.945, Tucker-Lewis Index=0.936, root mean square error of approximation=0.05, standardised root mean square residual=0.05). Figure 1 presents the latent factor constructs of the final model with standardised factor loadings on the paths. All loadings were statistically significant (p<0.001), and all measures had loadings >0.4 on each factor. Characteristics of the cognitive tasks comprising each factor are listed in table 2. All subsequent results were based on the cognitive composites.

**Table 2:** Summary of neuropsychological tests and outcome variables in each cognitive factor

Domain	Cognitive task	Outcomes	Range	Mean (SD)
Episodic memory	Logical Memory	Immediate recall total score	10–66	43.43 (9.03)
		Delayed recall total score	0–48	27.44 (7.01)
	Paired Associates	Immediate recall mean score	0–6	2.12 (1.41)
		Delayed recall mean score	0–6	1.43 (1.39)
	MoCA delayed recall	Delayed free recall	0–5	3.02 (1.55)
	Picture Sequence	Total raw score	0–31	10.37 (5.93)
	Hopkins Verbal Learning Test (HVLT)	Total immediate recall raw score	12–36	26.00 (4.49)
		Delayed recall (Trial 4) raw score	1–12	9.15 (2.11)
		Recognition discrimination Index Score	4–12	10.62 (1.44)
	Brief Visuospatial Memory Test - revised (BVMT)	Total immediate recall raw score	3–36	21.10 (6.42)
		Delayed recall raw score	1–12	8.66 (2.53)
Processing speed	Digit Symbol Substitution Test (DSST)	Total correct	24–78	47.11 (9.41)
	Trail Making Test, Part A	Completion time (s)	17–78	35.75 (10.16)
	Letter Comparison Test	Total correct mean score	3–14.5	8.75 (1.83)
Working memory	Spatial Working Memory task	3-Item accuracy	0.23–1	0.76 (0.13)
		4-Item accuracy	0.12–1	0.75 (0.12)
	N-Back Working Memory task	2-Back accuracy	0.21–1	0.81 (0.14)
	List Sorting Working Memory Test	Total correct	6–25	16.06 (2.58)
Executive function/ attentional control	Trail Making Test, Part B	Completion time (s)	29–300	77.37 (34.30)
	Dimensional Change Cart Sort (DCCS)	Computed score	3.25–10	7.76 (0.80)
	Stroop task	Incongruent response time	508.40–1848.08	926.48 (172.85)
	Flanker task	Computed score	5–9.56	7.63 (0.62)
Visuospatial	Spatial Relations Test	Accuracy	0–0.9	0.33 (0.19)
	MoCA clock draw*	Total score	4–10	8.60 (1.08)
	Matrix reasoning	Accuracy	0–1	0.48 (0.20)

Range column depicts the lowest and highest scores obtained by participants onfor each cognitive task.

* MoCA Cclock Ddrawing was scored using the Rouleau criteria (range 1–10)^79^ to provide a more comprehensive assessment of performance. Cognitive Assessment.

MoCAMontreal Cognitive Assessment

### Age and education associations with cognitive performance

As anticipated, older age was associated with poorer performance for all five cognitive domains (all p<0.001) (online supplemental figure S1), controlling for sex, site, education, and BMI. In addition, after controlling for age, sex, site and BMI, more years of education were associated with better performance for all cognitive domains (all p<0.001; online supplemental figure S1).

### CRF associations with cognitive performance

**Figure 2:**
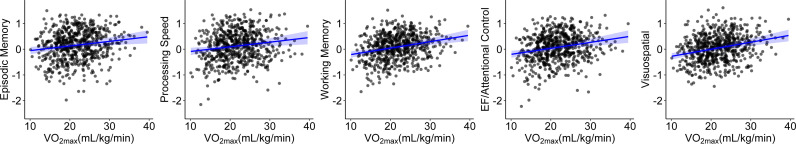
Associations between cardiorespiratory fitness (CRF) and cognitive performance in each domain. Higher CRF (VO_2max_) was significantly associated with better performance in all five cognitive domains. Solid blue represents best-fit line, shaded areas reflect 95% confidence intervals. EF, executive function.

Higher CRF was independently and positively associated with better cognitive performance for all five cognitive domains after adjusting for age, sex, site, education, *APOE4* carriage and BMI (figure 2; table 3). Associations with each cognitive factor persisted after additional adjustment for max RER ≥1.10 (yes/no), a physiological marker of achieving a maximal effort on the graded exercise test (all p<0.01). Associations also remained significant when adjusting for max RER as a continuous variable (all p≤0.01), and when controlling for whether or not participants achieved three of four criteria for maximal effort (all p<0.05).

**Table 3:** Results of regression analyses examining associations between cardiorespiratory fitness and cognitive factors

Predictors	Episodic memory				Processing speed				Working memory			
	B	β	t	p	B	β	t	p	B	β	t	p
Age	−0.025	−0.15	−3.821	<0.001	−0.044	−0.262	−6.699	<0.001	−0.033	−0.23	−6.016	<0.001
Sex	−0.338	−0.249	−6.078	<0.001	−0.151	−0.109	−2.674	0.008	−0.083	−0.069	−1.744	0.082
Education	0.072	0.259	6.835	<0.001	0.066	0.233	6.162	<0.001	0.07	0.284	7.69	<0.001
BMI	0.004	0.041	0.919	0.359	−0.002	−0.02	−0.442	0.659	0.008	0.081	1.847	0.065
*APOE4* carriage	−0.073	−0.053	−1.443	0.15	−0.043	−0.031	−0.844	0.399	−0.053	−0.044	−1.224	0.221
VO_2max_	0.018	0.148	2.945	0.003	0.018	0.146	2.928	0.004	0.025	0.234	4.8	<0.001
	Adjusted R^2^=0.158, p<0.001				Adjusted R^2^=0.163, p<0.001				Adjusted R^2^=0.203, p<0.001			

Predictors	Attentional Control				Visuospatial Processing			
	B	β	t	p	B	β	t	p
Age	−0.042	−0.263	−6.807	<0.001	−0.028	−0.186	−4.878	<0.001
Sex	−0.05	−0.038	−0.951	0.342	−0.047	−0.038	−0.95	0.342
Education	0.068	0.251	6.723	<0.001	0.081	0.316	8.568	<0.001
BMI	0.002	0.022	0.502	0.616	0.011	0.112	2.568	0.01
*APOE4* carriage	−0.042	−0.031	−0.867	0.386	−0.025	−0.02	−0.562	0.574
VO_2max_	0.023	0.198	4.015	<0.001	0.027	0.245	5.031	<0.001
	Adjusted R^2^=0.186, p<0.001				Adjusted R^2^=0.203, p<0.001			

*APOE4* carriage=non-carrier: 0; carrier: 1; sex=male:1, female: 0. Site was also included as a covariate in all models (data not shown).

BMIbody mass indexEFexecutive function

### Examination of moderators

We examined whether age, sex, education, *APOE4* carriage and factors related to the measurement of CRF moderated associations between CRF and cognitive performance. First, there was no significant age × CRF interaction for any cognitive domain (online supplemental table S1), indicating that the positive association between CRF and cognitive performance did not vary as a function of age in this sample of older adults.

**Figure 3:**
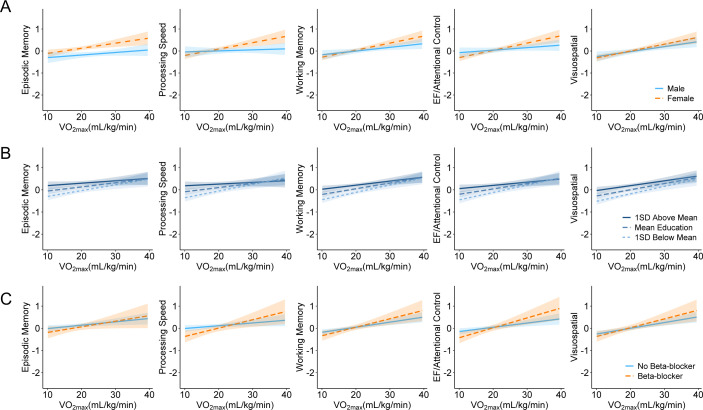
Associations between cardiorespiratory fitness (CRF) and cognitive factors as a function of sex, education, and beta-blocker use. Results from moderation models. (**A**) Relationship between CRF and cognitive factors as a function of sex (female, orange; male, blue). There was a significant sex × CRF interaction in the processing speed and executive function/attentional control domains. (**B**) Relationship between CRF and cognitive factors at different levels of education. There was a significant education × CRF interaction for processing speed and executive function/attentional control. For visualisation, each line represents the association between CRF and cognitive composites when all data points are at the mean (blue dotted line), 1 SD above (blue solid line), or 1 SD below (light blue dotted line) the mean years of education. (**C**) The relationship between CRF and cognitive factors as a function of beta-blocker use (blue, no beta-blocker use; orange, beta-blocker use). There was a significant beta-blocker × CRF interaction in the executive function/attentional control domain. Shaded areas reflect 95% confidence intervals. EF, executive function.

We found a significant sex × CRF interaction for processing speed (β=−0.447, t=−2.435, p=0.015) and EF/attentional control (β=−0.417, t=−2.302, p=0.022)(figure 3A), such that higher CRF was associated with better performance only among women. No significant sex × CRF interactions were found for the other cognitive domains (online supplemental table S2).

There was a significant education × CRF interaction for processing speed (β=−0.863, t=−2.378, p=0.018) and EF/attentional control (β=−0.759, t=−2.12, p=0.034)(figure 3B), such that higher CRF was associated with better performance among those with fewer years of education. No significant interactions were found for episodic memory, working memory or visuospatial processing (online supplemental table S3).

There was no significant main effect of *APOE4* carriage for any cognitive domain (all p>0.2; online supplemental table S4). Furthermore, the interaction terms of *APOE4* carriage × CRF on cognitive performance were not significant, indicating that the association between CRF and cognition did not significantly differ between *APOE4* carriers and non-carriers. Given evidence of a potentially protective effect of *APOE2* carriage on dementia risk,^48 49^ sensitivity analyses excluding APOE ε2/ε4 carriers (n=20) were conducted, and the results remained non-significant. Exploratory analyses testing a three-way interaction between CRF, sex, and *APOE4* carriage did not achieve significance in any domain (all p>0.35).

There was a significant beta-blocker × CRF interaction for EF/attentional control, such that the positive association between CRF and EF/attentional control was greater among those taking beta-blockers (β=0.305, t=1.989, p=0.047) (online supplemental table S5; figure 3C). Whether or not participants achieved three of four criteria for maximal effort did not moderate observed associations (all p>0.05). Sex and education interaction terms remained significant after additional adjustment for max RER, examined as both a continuous variable and dichotomised based on whether a max RER ≥1.10 was reached. The beta-blocker × CRF interaction was attenuated and marginal after adjusting for max RER as a dichotomous variable (β=0.297, t=1.955, p=0.051), but remained significant when adjusting for max RER as a continuous variable (β=0.304, t=1.997, p=0.046).

## Discussion

Consistent with our hypotheses, we found that higher CRF was associated with better cognitive performance across multiple domains susceptible to age-related cognitive decline.^50 51^ Sex, education and use of beta-blockers moderated associations in select cognitive domains, while higher CRF was beneficial regardless of age and *APOE4* carriage. These findings demonstrate the breadth of cognitive benefits associated with higher CRF, and highlight several key factors that might influence the relationship between fitness and neurocognitive health in older adulthood.

We found that CRF, which predicts the onset of numerous diseases and can be modified by regular exercise, was positively associated with cognitive performance across all domains assessed. We extend existing literature by examining cognitive function using a comprehensive neuropsychological battery and a factor analytic approach to reduce dimensionality and minimise methodological error associated with interpreting individual cognitive test scores.^52 53^ These findings also expand studies that have used proxy or surrogate measures of CRF^16^,^20^ and abbreviated or domain-specific cognitive assessments.^911^,^13 16^ For instance, a population-based study of 2500 older adults observed a positive association between fitness and cognitive performance, although used an equation-based estimate of CRF and measured cognition using a single cognitive task (digit symbol substitution task).^19^ Using latent factors reflecting multiple cognitive domains, we observed a broad, domain-general relationship of CRF with cognitive functioning.

The mechanisms underlying these associations are not fully understood and might occur across multiple levels of analysis.^54 55^ On a cellular level, mechanisms may include effects of aerobic fitness on cerebral blood flow, oxidative stress, synaptogenesis, neurotrophic factors, neurotransmitter systems, and others (see Stillman *et al* 54 55 for review). Additionally, changes to the macroscopic properties of the brain, including changes in grey matter morphology^22^ and white matter microstructure,^56^ might mediate the relationship between CRF and cognitive function. Psychosocial factors linked to CRF (eg, mood, fatigue, sleep) might also affect cognition,^54 55^ although further research is needed to better characterise these pathways.

Several demographic and genetic factors might moderate the association between CRF and cognitive function in older adulthood,^4 6 57^ which might explain some of the heterogeneity across studies that have failed to account for these moderators. However, knowledge of potential moderators is limited due to the use of restricted cognitive batteries, proxy measures of CRF and small sample sizes.^26^,^2858^ For example, cross-sectional studies employing a gold-standard CRF measure and comprehensive assessments of multiple cognitive domains have involved samples ranging from 31 to 120 older adults.^5^,^1014^ Large-scale studies better powered to evaluate putative moderators have largely employed proxy measures of CRF^16^,^20^ and brief cognitive batteries.^18 19 21 22^ This study overcame those limitations by using a comprehensive neuropsychological assessment along with a sample size much larger than most others in the field, which enabled a rigorous examination of moderators across multiple domains of cognitive functioning.

Sex significantly moderated CRF associations with processing speed and EF/attentional control, such that associations were positive and significant only among women in both domains. Few studies have examined sex differences in the relationship between CRF and cognition in older adulthood.^11 17 20^ Using an equation-based CRF estimate, one study of 315 older adults found no sex differences in associations with memory performance,^20^ while another observed that the positive relationship between CRF and memory performance was specific to men (n=25) but not women (n=40).^11^ Most prior work examining sex differences has focused on exercise behaviours, with some evidence suggesting that exercise efficacy might be greater in women in specific cognitive domains.^59^,^63^ Consistent with the current findings, a meta-analysis of exercise interventions reported greater improvements among women relative to men in the domain of executive function, whereas no sex differences were observed in the domains of episodic memory or visuospatial function.^59^ The mechanisms are currently speculative but might include sex steroid hormones and sex differences in pathways that are influenced by fitness and exercise behaviours, including synaptogenesis, brain-derived neurotrophic factor signalling and hypothalamic-pituitary-adrenal axis regulation.^25 64 65^

In addition, several studies suggest that higher CRF might be protective against cognitive impairment (eg, Alzheimer’s disease), even in the presence of risk factors for cognitive decline.^3 11 14 16^ The present findings support this notion in several ways. First, fewer years of education are predictive of greater age-related cognitive decline and dementia risk.^66^ Importantly, we found that the positive association between CRF and cognitive performance was amplified among those with fewer years of education in select cognitive domains. These findings, if replicated, suggest that the presence of one protective factor (eg, higher CRF) could attenuate the lack of another (eg, few years of education). Second, both greater age and *APOE4* carriage are associated with elevated risk of Alzheimer’s disease,^67^ but we found that the positive association between CRF and cognitive performance did not vary as a function of age or differ between *APOE4* carriers and non-carriers. These data indicate that those at elevated risk for cognitive decline—*APOE4* carriers and individuals at the higher end of the age spectrum—showed a similar degree of fitness-related cognitive benefit. This aligns with work demonstrating positive associations between CRF and cognition independent of age and *APOE* status,^3 16^ and highlights the promising potential of CRF to preserve cognitive health even among those at elevated risk of decline.

It is notable that most studies examining APOE as a moderator have focused on exercise behaviours and have yielded mixed results.^23 68^ While some data suggest that the favourable effects of physical activity on cognitive decline and dementia risk might be specific to non-carriers,^69 70^ other studies report greater cognitive benefits among *APOE4* carriers^71 72^ or find no difference between *APOE4* carriers and non-carriers.^23 73 74^ Demographic and exercise training factors might influence the relationship between APOE status and physical activity, contributing to study heterogeneity. A recent meta-analysis of eight intervention studies found that differences in exercise efficacy between *APOE4* carriers and non-carriers varied based on exercise intensity and intervention duration.^68^ Specifically, *APOE4* carriers and non-carriers showed similar cognitive benefits from briefer interventions and low–moderate intensity activity, while significant differences in cognitive gains were observed in favour of *APOE4* non-carriers in interventions involving >50 sessions and higher-intensity activity. There are also complex APOE genotype × sex interactions such that female *APOE4* carriers have a greater risk of developing Alzheimer’s disease than male carriers.^35^ Thus, potential APOE-related differences in response to physical activity might vary by sex.^75^ Exploratory analyses assessing potential sex differences in APOE moderation were non-significant, although the study might not be adequately powered to detect three-way interaction effects. Additional studies are needed to clarify how these factors, and others, might influence exercise efficacy in relation to APOE carrier status.

Finally, there is significant variability in how CRF is studied across the field, and especially in older adults, in whom beta-blockers, orthopaedic limitations or other health conditions might impair the ability to reach a physiological maximum.^15 76^ Even in this study, we found variability in achieving the criteria for attaining VO_2max_, and observed an inverse association between achieving a VO_2_ plateau and VO_2max_. The potential contributors to this association warrant further examination in older adults and might inform future studies of CRF and its association with ageing and cognition. We also found a moderating effect of beta-blocker use in the EF/attentional control domain, such that the positive association between CRF and cognitive performance was amplified among those taking beta-blockers. This might be attributable to cardiometabolic conditions in participants requiring beta-blocker medication, and highlights the importance of maintaining higher CRF in the context of these conditions to preserve EF/attentional control. Additionally, we found that associations between VO_2max_ and cognitive performance persisted after adjustment for physiological capacity (assessed via RER), suggesting that both physiological and volitional components related to the measurement of VO_2max_ might drive associations with cognition in older adulthood.

### Limitations

The primary limitation of the study is the cross-sectional approach, which does not permit causal inferences. Results from the IGNITE intervention will shed additional light on the causal importance of manipulating VO_2max_ for cognitive improvements resulting from engagement in exercise. Additional insight might be gained by prospective studies to examine trajectories of change and the role of putative moderators. Although we obtained a comprehensive cognitive battery, not all cognitive processes were represented (eg, language). Participants were considered relatively inactive at the time of enrolment. This might have restricted the range of fitness levels observed, but our results suggest that even small differences in fitness can have an important relationship with multiple aspects of cognition. Additionally, the sample was comprised of cognitively healthy, sedentary older adults capable of participating in an exercise intervention. Other studies are needed to replicate our findings across varied populations, including a greater proportion of men, physically active older adults, individuals with cognitive impairment, various levels of education and a broader age range to capture those in the ninth and tenth decades of life. Although the sample demographics reflected the population demographics of each site, including 24% of individuals from racial and ethnic minority groups, achieving census-based recruitment targets might not be sufficient to make broader statements about the generalisability of results to diverse populations. Future efforts to maximise representation of diverse populations will enhance understanding of these relationships.

Finally, despite the study findings showing associations between CRF and cognitive outcomes, even when accounting for several factors that might influence VO_2_ measures, there are general limitations to how CRF is measured that might result in some participants not achieving a true physiological maximal effort, which can contribute to variability in this measurement. For instance, in this study, more than 50% of participants did not reach the RER threshold (≥1.10); however, 78% reached three out of four criteria for maximal effort.^31^ Although the results persisted after adjusting for these factors, future studies need to account for these differences when reporting the association between CRF and cognitive outcomes in older adults. Additionally, while we implemented a standard approach to CRF assessment (graded exercise test), there are various limitations of the different types of protocol used to measure CRF when the desire is to achieve a true physiological maximal effort (eg, RER threshold, selected speed), which could also be contributing to variability across the literature. While we performed comprehensive neuropsychological testing with consensus adjudication for those with mild cognitive impairment (MCI) and dementia, given known limitations of cognitive testing and the often variable definitions of MCI, it is possible that some participants were included who could be near the MCI range. Though beyond the scope of the study, an examination of cellular and molecular mechanisms would offer insight into potential pathways linking CRF to cognitive and brain health in older adulthood.

### Clinical implications

CRF, which can be modified by structured exercise training programmes,^77 78^ was positively related to cognitive benefits across multiple domains susceptible to age-related decline.^50 51^ These data emphasise CRF as an important health factor and therapeutic target for preserving multidomain cognitive functioning in late adulthood. These findings also clarify the moderating role of several factors, which might help to inform public health recommendations and the development of individualised fitness prescriptions to optimise cognitive health in older adulthood.

## supplementary material

10.1136/bjsports-2024-108257 online supplemental file 1

10.1136/bjsports-2024-108257 online supplemental file 2

## Data Availability

Data are available upon reasonable request.
