# Supplementary material for: Cardiorespiratory fitness is associated with cognitive function in late adulthood: baseline findings from the IGNITE study
Source: Br J Sports Med. 2024 Dec 10;59(3):e108257. doi: 10.1136/bjsports-2024-108257 (PMC11790366; doi:10.1136/bjsports-2024-108257)
Supplement: online supplemental file 1 [file bjsports-59-3-s001.pdf]

CHAPTER 1

IGNITE PROTOCOL

1.0 SUMMARY:

Despite the ubiquity of normal age-related cognitive decline there is an absence of effective approaches for improving neurocognitive health. Fortunately, moderate intensity physical activity (PA) is a promising method for improving brain and cognitive health in late life, but its effectiveness remains a matter of continued skepticism and debate because of the absence of a Phase III clinical trial. Here we propose a Phase III multi-site randomized clinical trial called IGNITE (Investigating Gains in Neurocognition in an Intervention Trial of Exercise) to more definitively address whether exercise influences cognitive and brain health in cognitively normal older adults. We have proposed a 12-month, multi-site, randomized dose-response exercise trial (i.e., brisk walking) in 639 cognitively normal adults between 65-80 years of age. Participants will be randomized to a (a) moderate intensity aerobic exercise condition at the public health recommended dose of 150 minutes/week (N=213), (b) a moderate intensity exercise condition at 225 minutes/week (N=213), or (c) to a stretching-and-toning control condition for 150 minutes per week (N=213). Participants will meet 3 days/week for site-based exercise and do home-based activity on two more days of the week for 12 months. A comprehensive state-of-the-science battery of cognitive, MRI, amyloid imaging, physiological biomarkers, cardiorespiratory fitness, physical function, and quality of life measures will be assessed at baseline and after completion of the intervention.

We have assembled a highly creative, productive, and interdisciplinary team with a long history of collaboration and experience conducting exercise interventions in older adults to test the following aims:

Aim 1: Cognitive Enhancement: using a comprehensive neuropsychological battery and the NIH Toolbox, we will test whether a 12-month moderate intensity exercise intervention improves cognitive performance in older adults and (b) test whether the improvements occur in a dose-dependent manner.

Aim 2: Brain Augmentation: we will test whether a 12- month PA intervention augments MRI markers of brain health and whether these changes happen in a dose- dependent manner.

Aim 3: Biomediators: we will test the hypothesis that cardiorespiratory, inflammatory, and neurotrophic changes mediate improvements in brain and cognition.

Aim 4: Moderators: we will examine subgroups (i.e., individual differences) that attenuate or magnify the effect of the intervention on cognitive, brain, and physiological systems to better understand the factors that predict ‘responders’ versus ‘non-responders’ to the intervention. We will explore three categories of variables: (1)

demographic (e.g., age) (2) genetic (e.g., APOE), and (3) baseline Aβ burden.

Exploratory Aims: We will explore (a) whether baseline brain health metrics predict adherence and compliance to 12-months of PA, and (b) the utility of multi-modal brain imaging analytical approaches to more comprehensively understand the effects of PA on the aging brain. The results from this trial could transform scientific-based policy and health care recommendations for approaches to improve cognitive function in cognitively normal older adults.

1.1 BACKGROUND AND RATIONALE:

A rapidly changing demographic landscape will result in a doubling of the population 65 years and older, which will reach 71 million by 2050[2]. Such figures have important medical, societal, and economic implications because these extended years are likely to be accompanied by chronic disease, disability, cognitive decline, and compromised quality of life[4]. Cognitive losses, including normal age-related declines, remain one of the most salient and feared consequences of aging[5]. Physical activity (PA) is one of the more promising and cost-efficient methods to improve neurocognitive function, but there continues to be considerable skepticism about its effects given the absence of a Phase III clinical trial. In fact, a recent 2015 Cochrane Review of 12 randomized trials concluded "We found no evidence in the available data that aerobic physical activities, including those that successfully improve cardiorespiratory fitness, have any cognitive benefit in cognitively healthy older adults."[6]. In stark contrast, other meta-analyses have arrived at the opposite conclusion: That PA is effective at enhancing cognitive function[7-10]. In fact, the Institute of Medicine issued a statement in April 2015 that there is sufficient scientific evidence to recommend PA to cognitively healthy older adults to maintain and improve cognitive function[11]. These mixed messages are being transmitted to the public and scientific community, creating risk for confusion, skepticism, and frustration. The lack of scientific consensus and continued debate can be put to rest by a RCT designed to conclusively determine whether PA improves neurocognitive outcomes. The results of our study could have an immediate impact on public policy and health care recommendations in addition to advancing the state-of-the-science in cognitive aging, neuroscience, and epidemiology.

We focus here on cognitively normal older adults instead of individuals with mild cognitive impairment (MCI) or Alzheimer's disease (AD). It is estimated that ~30% of individuals >65 yrs meet criteria for dementia or MCI, indicating that "normal" cognitive decline is far more prevalent than impairment[1]. Normal age-related cognitive changes are preceded by changes in the structure and function of brain regions including those supporting memory (hippocampus) and executive functions (PFC)[12-19] and it is estimated that 20-40% of cognitively normal adults have evidence of β-amyloid (Aβ). Is PA an effective approach to improve neurocognitive function in cognitively normal older

adults? What are the mechanisms? Are the current dose recommendations sufficient for enhancing cognitive outcomes? Why do some people respond to exercise and others do not? Is there a 'point of no return' such that those with evidence for Aβ do not benefit as much as adults without Aβ? The absence of answers to these questions is limiting widespread adoption and prescription of exercise to ameliorate cognitive problems in late life; and these are the questions we aim to answer.

1.2 EVIDENCE FAVORING PHYSICAL ACTIVITY:

Moderate intensity aerobic exercise has emerged as a promising low-cost method for improving neurocognitive function in older adults. Animal studies have conclusively shown that exercise enhances brain vascularization, increases neurogenesis, and influences gene expression in addition to improving learning and memory[20-24]. In humans, physical inactivity is a major risk factor for dementia and contributes to 21% (over 1.1 million) of AD cases in the US[25], and also predicts normal age-related losses in cognitive function[26]. Meta-analyses with >30,000 participants suggest that PA may reduce the risk of cognitive impairment by 40% or more[26]. These results are complemented by cross-sectional studies that have demonstrated that more active and higher fit adults outperform their more sedentary peers across several cognitive domains[27]. Observational and prospective studies, however, are inherently limited in their ability to determine causal associations between PA and cognition. Causal evidence comes from several RCTs, which demonstrate that moderate intensity exercise for ~6-months is effective at improving executive function, processing speed, and memory[8,28-30]. For example, in a 6-month moderate intensity RCT, we demonstrated improvements in executive function in 124 cognitively normal older adults[29]. Several meta-analyses of RCTs suggest that PA is effective at improving cognitive performance in older adults[7-10] with small-to-moderate effect sizes ranging from .10 to .70 depending on the outcome and duration of PA.

In addition to studies on cognition, evidence from neuroimaging studies suggests that PA alters both brain morphology and function in a regionally specific manner with the PFC and hippocampus most consistently affected[31]. For example, we found that a moderate intensity exercise RCT in 120 cognitively normal older adults increased the size of the hippocampus but not the caudate nucleus or thalamus[32] (also see[33,34]). We also found that a 6-month moderate intensity RCT increased the volume of the PFC and anterior cingulate cortex (ACC), but not the occipital cortex[35]. Similar specificity has emerged from task-evoked activation and functional connectivity. For example, work from our group and others have shown that moderate intensity PA for 12- months increased task-related brain activity in the PFC[36],[37],[38], and functional connectivity between PFC, ACC, and hippocampal nodes[39],[40]. In sum, there is considerable evidence

from animal studies, prospective longitudinal studies, and RCTs that moderate intensity exercise may be capable of improving neurocognitive function in a domain and regionally specific manner.

1.3 EVIDENCE DISPUTING PHYSICAL ACTIVITY:

Despite the wealth of human and rodent data demonstrating promising effects of PA on brain and cognition, other studies fail to find significant effects and contribute to the continued skepticism about whether PA is effective for improving cognition. For example, in a 12-week RCT in 126 older adults, both exercising and control groups showed equivalent improvements in cognition[41]. In another 14-month RCT with 101 cognitively normal older adults, there were no significant improvements in cognitive outcomes relative to the control group[42].Finally, the largest and longest RCT of physical activity to date (The LIFE study) reported no significant improvements in cognition for the exercise compared to the control group, though an analysis of subgroups revealed a significant improvement in executive functioning in the oldest and most physically impaired subgroups[43]. Several meta-analyses of RCTs have also argued that there is currently insufficient evidence that PA improves cognitive performance in dementia[44] or in MCI[45], and a recent 2015 Cochrane Review argued that there is insufficient evidence that exercise is effective at improving cognitive performance in cognitively normal adults[6]. The neuroimaging literature is less equivocal about the effects of exercise on brain morphology and function, but there have also been fewer RCTs that have incorporated neuroimaging measures with none examining Aβ [32-34,36,37,39,40,46-49].

1.4 INNOVATION:

It is important to appreciate the novelty of our proposed study in the context of recent or on-going exercise interventions. A currently funded study is examining 12-months of exercise on cognitive function and whole-brain volume in 300 individuals with amnestic MCI (PI: Baker; AG010483). The LIFE study was designed to examine effects of exercise on physical disability and did not include comprehensive cognitive or brain imaging measures. A recently funded trial (PI: Lenze; AG049369) is comparing the effectiveness of 6-months of exercise to a mindfulness-based intervention. The Action Seniors! Study is examining the effects of exercise on risk for falls while another (e.g., Brain in Motion; n=250) is testing cerebrovascular pathways of ~6 months of exercise with only a small percent receiving brain imaging. In sum, our proposed study (n=639) will be the largest of its kind, unique in its focus on cognitively normal adults, distinct in its design and implementation and detailed objective measurement of physical fitness and activity, its manipulation of dose of exercise and rigorous measurement of potential

mechanisms, and its cutting-edge analytical approaches. The Phase III nature of our study will lead to immediate public health implications for normal cognitive aging that is lacking in previous and on-going studies.

Our primary goal is to conduct a study that will transform scientific-based policy and health care recommendations for improving cognitive function in older adults. We have assembled a highly productive and interdisciplinary team of investigators with a long history of collaboration to provide answers to important unsettled scientific questions that have critical public health consequences:

(1) For the first time we will test, in a multi-site randomized Phase III clinical trial, whether moderate intensity exercise is effective at improving cognitive function in cognitively normal older adults.

(2) For the first time, we will use cutting-edge MRI sequences and analytical approaches (e.g., graph metrics of intrinsic functional brain connectivity) to determine whether exercise improves brain health.

(3) For the first time, we will examine dose-response effects of exercise and determine whether the current recommended guidelines for PA is sufficient for improving neurocognitive function.

(4) For the first time our proposed sample size will allow us to assess individual differences (e.g., baseline Aβ load) in treatment efficacy.

(5) For the first time our sample size will be large enough to test several competing hypotheses about the physiological mediators of exercise on cognitive and brain systems.

(6) For the first time, we will have the capability and expertise to execute latent growth curve, structural equation, and hierarchical modeling to examine the effects of exercise on statistically-derived latent constructs of cognitive function, to evaluate the amount of variance in cognitive improvements explained by imaging and biomediators, and to evaluate the rate of change in cognitive outcomes over a 12-month period.

1.5 PRELIMINARY DATA:

1. Effects of exercise on cognitive function: Our group was one of the first to demonstrate the favorable effects of a 6-month aerobic exercise RCT on cognitive outcomes[8,29]. In one study, 124 sedentary adults 60-75 yrs of age were randomly

assigned to either aerobic exercise or a stretching and toning control condition. Compared to the control, aerobic exercise improved executive functions[29]. In a meta-analysis of 18 RCTs in older adults, we found that exercise broadly influenced cognitive function (Hedge's g=.47), but improved executive function more than other cognitive domains (Hedge's g=.67), with the largest effect sizes for study durations of 6 months or longer[8]. These encouraging effects have now been replicated by our group and others[30,32,50,51,52]. Implications for this application: We have significant experience conducting PA interventions.

Our results demonstrate that >6-months of PA improves cognitive function and that we have been successful at recruiting and retaining older adults into PA intervention trials.

2. Effects of exercise on brain outcomes: Our research group has published >70 papers on the effects of exercise and fitness on brain morphology, white matter integrity, resting state connectivity, and task-evoked functional MRI activity. In 120 cognitively normal adults between 60-80 yrs of age (N=60/group), moderate intensity exercise was effective at increasing hippocampal volume, which was correlated with improvements in spatial memory. Exercise also increased volume of the PFC and ACC in 59 (N=~28/group) cognitively normal adults[35]. In addition to brain morphology, changes in cardiorespiratory fitness resulting from an exercise RCT were correlated with increased white matter integrity in PFC and temporal lobes in 70 cognitively normal adults[49]. Our results also extend to resting-state connectivity[39,40,61] and task-evoked activation[36]. For example, in 65 cognitively normal older adults exercise increased functional connectivity[39] and these changes were correlated with improvements in executive function, memory, and increases in serum BDNF and IGF-1[50]. Implications for this application: Our team is well-versed in the methods, challenges, and analytical approaches for examining MRI outcomes in PA interventions. We have been successful in coordinating neuroimaging efforts across sites and maintaining participants in MRI studies that are >1 year in duration.

3. Dose response effects of exercise: Our team conducted the first dose-response PA study. We randomized 101 cognitively normal older adults to control, 75, 150, and 225 minutes/week of moderate-intensity aerobic exercise for 26-weeks. Cognitive outcomes were latent residual scores derived from a battery of 16 cognitive tests. Analyses restricted to per-protocol demonstrated that the domain of 'Attention' improved equivalently across all exercise groups compared to controls and a dose-response relationship was present for 'Visuospatial Processing' with 225 minutes showing greater effects than 150 minutes. Implications for this application: We have the expertise to successfully execute a dose-response PA study and that cognitive improvements may differ as a function of dose.

1.6 STATISTICAL ANALYSIS:

Sample Size Justification: Calculations were performed to determine the statistical power for the target sample size of 639 (N=213/condition). We estimate power based on the linear contrasts resulting from a repeated measures model. Based on a clinically conservative effect size of .50, N=91/group is necessary to reach 80% power at p<=0.05/2=.025 to limit inflation of Type I error due to multiple testing of two-sided hypotheses using Bonferroni correction. Even with 20% attrition, we will be sufficiently powered to test our primary hypotheses. For dose effects, we estimate an effect size of .30 from our preliminary results (Vidoni et al., 2015). As such, N=176 per group is sufficient to detect differences in each cognitive outcome between groups with 80% power. Effect sizes from neuroimaging data (0.40-0.50) indicate that we will be sufficiently powered to test for differences in volume, functional connectivity, white matter, and task-evoked activity. In sum, our planned sample size of 639 (N=213 per group) will be sufficient for allowing us to test our primary hypotheses with enough residual power to further examine statistical mediators and individual differences. Preliminary steps: Prior to hypothesis testing, all data will be examined to determine: 1) frequency distributions for missing data and out-of-range values; 2) normality and internal consistency of subscales; and, 3) association between variables that may be highly correlated to guard against multicolinearity, which would inflate standard errors and make estimation unstable. We will also examine the distribution of factors between groups to assess whether covariate-adjustment is required. Missing data: All outcomes will be tested using an intent-to-treat (ITT) framework. Sensitivity will be explored using adherence to the intervention (e.g., per protocol). We will report reasons for dropouts and explore missing data mechanisms. To examine whether missingness is unrelated to other observed measurements (missing completely at random, MCAR) or to the observed measurements only (missing at random, MAR), testing for completely random dropouts will be carried out. Additional sensitivity analyses will be performed to test for informative dropouts. We will apply pattern-mixture models by stratifying our data by dropout patterns and fitting separate regression models to strata.

Testing Primary Aim:

The primary endpoint will be a composite score of executive function. More specifically, we are predicting that there will be a significant exercise-related improvement in executive function over the course of the 12-month period (i.e., Time x Group interaction term for executive function for the exercise arms compared to the control arm). Changes in composite scores of other cognitive domains including episodic memory and processing speed will be considered secondary. Our analysis plan focusing on a composite of executive function indicates that our primary outcome is not a measured outcome (e.g., Stroop task performance) but rather a latent factor composed of performance on several tasks, which is considered to more accurately represent the cognitive domain (since performance on any one task is not a reliable representation of any cognitive domain). The latent factors and loadings for executive function will be determined based on a confirmatory factor analysis using baseline data from the following measures:

1. Synonym / Antonym
2. Logical Memory
3. Spatial Relations
4. Paired Associates
5. Matrix Reasoning
6. Dimensional Card Sort
7. List Sort Working Memory
8. Picture Sequence
9. Flanker
10. MOCA
11. Trail Making A & B
12. Spatial Working Memory
13. Cohen's Relational Memory
14. Hopkins Verbal Learning Test (HVLT-R)
15. Stroop task
16. Task Switch
17. Letter and Category Fluency test
18. The Iowa Gambling Task
19. Brief Visuospatial Memory Test-Revised BVMT
20. Digit Symbol Substitution Test
21. Letter Comparison

Any missing cognitive data at baseline could influence the formulation of the latent factors from the factor analysis. To avoid this potential concern, we will conduct an analysis using only the collected baseline data to create the latent factors. But, then, we will conduct a secondary sensitivity analysis by imputing missing values in each of the outcomes for the missing baseline data and use these values for creating the factors scores. Only baseline data will be used for imputation and these data will be used for repeating the factor analysis. Then, we will compare the factor analysis results with and without missing value imputation.

By creating the latent factors based on data collected at baseline we will not be introducing intervention-related biases in the creation of the factors since the factors would be determined by data collected before randomization. However, we also plan to conduct post-hoc confirmatory factor analyses on the data collected at the follow-up assessments to determine consistency in the factor loadings across measurement time points. But, the results from this post-hoc confirmatory factor analysis will not be used for the modification of the baseline factor analysis results and will not influence the testing of the primary aim. We propose to test our primary hypothesis using a general linear mixed model (GLMM) approach because we will have three assessments of cognitive function over the course of the trial (baseline, 6-months, 12-months). The GLMM model will include both random intercept and random slope for individual participants and a treatment-by-time interaction as a fixed effect. This technique will allow us to model the changes in the latent factor score for executive function as a function of both time and group while also including other potentially confounding

variables (e.g., site) in the model. GLMM modeling of repeated assessments is a popular approach in clinical and longitudinal studies because it allows for random changes in measurement error and takes into account both within and between subject correlations and estimates the average growth curve across subjects and subject-specific growth curves.

1.7 PROCEDURES:

Before the collection of baseline data, all staff will have had ethics training and appropriate certification of research training modules. All staff will also be a part of an on-site training certification to ensure that all data collection practices will be done in the same way across all sites.

1.7.1 SCREENING:

During telephone screening interview information about participant's current exercise status will be collected and participants that engage in more than 20 minutes of structured exercise per week will be considered ineligible. During this phone screening, numerous medical history questions will be asked to verify subject meets inclusion criteria as no medical record retrieval is being used. Information about safety to exercise will be obtained over the phone (e.g., history of falls) to ensure that exercise will not be dangerous for the subjects. Finally, information regarding metal in the body for MRI safety will obtained, this includes information about any metallic objects in their body. Subjects will be required to have a primary care physician that study staff member will contact to obtain documentation that they are physically safe to be a part of a monitored exercise program. A letter will be sent to each participant's primary care physician (PCP) asking in their medical opinion, if they feel it is safe for their patient to participate in the trial. A slightly different version of the letter will be sent to those with Type II Diabetes to verify Hba1C is below 10 in order to meet inclusion criteria along with opinion it is safe for subject to participate in the trial.

1.7.2 INCLUSION AND EXCLUSION CRITERIA:

Inclusion Criteria:

- Men and women 65 – 80 yrs.
- Ambulatory without pain or the assistance of walking devices
- Able to speak and read English
- Exercise level less than 3 days per week of <20 minutes per day
- Medical clearance by PCP
- Living in community for duration of the study
- Reliable means of transportation

• No diagnosis of a neurological disease
• Eligible to undergo MRI

- **Exclusion Criteria:**
  • Current diagnosis of a DSM-V Axis I or II disorder including Major Depression
  • History of major psychiatric illness including schizophrenia (not including general anxiety disorder or depression)
  • Current treatment for cancer – except non-melanoma skin
  • Neurological condition (MS, Parkinson's, Dementia, MCI) or brain injury (traumatic or Stroke)
  • Type I Diabetes or uncontrolled Type II Diabetes defined as Insulin dependent or Hba1C greater than 10
- Current alcohol or substance abuse
- Current treatment for congestive heart failure, angina, uncontrolled arrhythmia, DVT or other cardiovascular event
- Myocardial infarction, coronary artery bypass grafting, angioplasty or other cardiac condition in the past year
- Regular use of an assisted walking device
- Presence of metal implants (e.g., pacemaker, stents, joint replacement) that would be MR ineligible
  • Claustrophobia
  • Not fluent in English
  • Not medically cleared by PCP
  • Engaging in >20 minutes on 3 days or more of moderate intensity physical activity per week
  • Travelling consecutively for 3 weeks or greater during the study
  * PET scan within past 365 days (Temporary exclusion)

1.7.3 BASELINE TESTING:

There are multiple outcome measurement sessions at baseline (6 for the Pittsburgh and Kansas sites and 5 for the site in Boston). There is an 8-week maximum window to complete these outcome measures. This 8-week window starts the day the informed consent is signed at session 1.

Session 1: Participants will come to the laboratory and complete a battery of neuropsychological tests (see below for a description of the tests). Compliance with the test instructions will be monitored by a certified instructor and neuropsychologist. Participants will be allowed to take breaks between tests on a regular basis to help reduce fatigue. After completion of the session, the tests will be scored at the coordinating center and a panel of neuropsychologists will

adjudicate each subject to determine if (based on the neuropsychological tests) the subject should be considered ‘demented’, ‘mildly cognitively impaired’, or ‘cognitively normal’. Only cognitively normal subjects will be considered eligible to continue in the study so any subject that is considered to have dementia or mild cognitive impairment will be excluded at this point and referred to their personal physician for further testing and follow-up. Upon completion of this session, participants will be given a packet of questionnaires to complete and return during session 2.

Session 2: The second visit will also consist of cognitive and neuropsychological tests that are collected as outcome measures (see below for a description of the tests). All of the tasks will be administered at baseline, 6-months and 12-month unless otherwise noted. The following tests will be completed during these two cognitive sessions:

The Virginia Cognitive Aging Project Battery is a standardized paper-and-pencil neurocognitive battery that assesses attention, memory, and processing speed. The IGNITE trial will be using the following tests from this battery:

- Synonym / Antonym (12 minutes) Participant is asked to select the best synonym or antonym of the target word.

- Logical Memory (10 minutes) Number of idea units recalled across three stories.

- Spatial Relations (12 minutes) Determine the correspondence between a 3-D figure and alternative 2-D figures.

- Paired Associates (3 minutes) Number of response terms recalled when presented with a stimulus term.

- Matrix Reasoning (12 minutes) Participant determines which pattern best completes the missing cell in a matrix.

The NIH Toolbox will also be utilized and several tests from the cognitive portion will be administered to subjects at all three time points (Baseline, 6-months, and 12-months), specifically the following tasks:

- Dimensional Card Sort: (6 minutes) A task that measures cognitive flexibility and attention. Two target pictures are presented that vary along two dimensions (e.g. shape and color). Participants are asked to match a series of bivalent test pictures (e.g. yellow balls and blue trucks)

to the target pictures, first according to one dimension (e.g. color) and then, after a number of trials, according to the other dimension (e.g. shape). The relevant dimension for sorting is indicated by a cue word (e.g. “shape” or “color”) that appears on the screen for all participants.

- List Sort Working Memory: (10 minutes) This task assesses working memory and requires the participant to recall and sequence different visually and orally presented stimuli. Pictures of different foods and animals are displayed with both an accompanying audio recording and written text that name the item. The participant is asked to say the items back to the examiner in size order from smallest to largest.

- Picture Sequence: (5 minutes) In this measure of episodic memory, sequences of pictures objects and activities are presented in a particular order. The participants are asked to reproduce the sequence of pictures that is shown on the screen.

- Flanker: (4 minutes) This is a measure of inhibitory control and attention. The task requires the participant to focus on a particular stimulus while inhibiting attention to the stimuli flanking.

MOCA (Montreal Cognitive Assessment): was designed as a rapid screening instrument for mild cognitive dysfunction. It assesses different cognitive domains: attention and concentration, executive functions, memory, language, visuoconstructional skills, conceptual thinking, calculations, and orientation. Time to administer the MoCA is approximately 10 minutes. The total possible score is 30 points; a score of 26 or above is considered normal. This task is only administered at baseline.

Trail Making A & B: This task measures processing speed and executive control. Both parts of this test consist of 25 circles distributed over a sheet of paper. In Part A, the circles are numbered 1-25, and the subject is instructed to draw lines to connect the numbers in ascending order. In Part B, the circles include both numbers (1-13) and letters (A-L); as in Part A, the patient draws lines to connect the circles in an ascending pattern, but with the added task of alternating between the numbers and letters (i.e., 1-A-2-B-3-C, etc.). The task is discontinued if the subject has not completed both parts after five minutes have elapsed.

Spatial Working Memory (SWM) measures spatial memory functions and takes about 10 minutes. It requires that subjects attend to and retain the location of several dots presented simultaneously on a computer display. They are

requested to press buttons on a keyboard that correspond to whether a probe dot appeared in one of the same locations as the previous dots.

Cohen's Relational Memory test is a computerized assessment related to relational/episodic memory and hippocampal function. The computer screen will display abstract objects (line drawings) in a certain configuration on the screen for a specified length of time, after which the objects disappear briefly and then reappear aligned at the top of the screen. The participant is asked to study each configuration, then click and drag the objects in order to move them to the previously displayed configuration as accurately as possible. The subject advances through multiple trials of this task, total task completion requires approximately 10 minutes.

Hopkins Verbal Learning Test (HVLT-R) measures immediate memory span, provides a learning curve, reveals learning strategies or the absence, and short-term and longer term retention. Subject has 3 trials to learn and then asked to recall the items after a 20-minute delay period.

The Stroop Task measures selective attention, cognitive flexibility and processing speed and is used as a tool in the evaluation of executive function. This task is administered on a computer and the participants are asked to respond to the colors of the ink for printed words on the display as quickly as they can while ignoring the meaning of the word. The task takes less than 10 minutes to complete.

A Task Switch paradigm in which stimuli are presented on a computer display and participants are asked to response to whether the number is greater than or less than 5 (task 1) or to respond to whether the number is odd or even (task 2). It takes approximately 10 minutes to administer and is considered to be a measure of executive function.

Letter and Category Fluency test: In this task, subjects are given a letter of the alphabet (F, A, S). In one minute, they must list as many items as possible that begin with that letter. In the category task, they are given a category (animals) and must name as many items in that category as possible in one minute.

The Iowa Gambling task simulates real life decision making. Participants are presented with 4 virtual decks of cards on a computer screen. They are told that each time they choose a card they will win some game money. Every so often, however, choosing a card causes them to lose some money. The goal of the game is to win as much money as possible. The task takes approximately 10 minutes to administer.

Brief Visuospatial Memory Test-Revised BVMT is a measure of visuospatial

memory. Participants are asked to recall visuospatial information and then after a 25-minute break retrieve the information provided during the study phases.

Wechsler Test of Adult Reading (WTAR) estimates pre-morbid intellectual functioning (approximate intelligence). This test takes approximately 5 minutes to administer and will only be administered at the baseline testing session.

Digit Symbol Substitution Test (DSST) Digit Symbol Substitution asks participants to match symbols with their corresponding digit. It consists of 9 digit symbols matched with their corresponding numerical digit and only takes approximately 3-4 minutes to administer.

Letter Comparison. In this task participants are asked to determine whether a series of letters is the same as another series of letters. They are asked to do this as quickly as they can. This task takes less than 5 minutes to administer.

The following questionnaires will be completed either during the outcome sessions or taken home by participants to complete. They are administered at baseline, 6-months, and 12-months:

Demographics, Health History, and Lifestyle:
- Health History
- Medication List
- MacArthur SES

Subjective Memory and Cognition:
- Abbreviated Cognitive Function Index (CFI)
- PROMIS Applied Cognition: Abilities
- PROMIS Applied Cognition: General Concern
- Everyday Cognition (ECOG)

Intellectual Engagement:
- Work Design (WDQ)
- Florida Cognitive Activity Scale

Diet and Nutrition:
- Diet History (NCI)

Quality of Life:
- Satisfaction with Life Scale (SWLS)
- Health Survey Short Form (SF-36)

Pain, Fatigue, Physical Symptoms:
- McGill Pain
- Fatigue Symptom Inventory (FSI)

Social Support:
- Social Goal Importance
- Exercise Group Social Provisions Scale (EXSPS)
- Social Provisions Scale (SPS)
- Sheldon Cohen's Social Network Index

Self-Esteem:
- Physical Self-Perception Profile (PSPP)
- Rosenberg Self-Esteem Scale (RSE)

Self-Efficacy:
- Barriers Self-Efficacy Scale (BARSE)
- Exercise Self-Efficacy (EXSE)
- Lifestyle Self-Efficacy (LSE)

Self-Regulation:
- Physical Activity Self-Regulation Scale (PASR-12)

Mood / State / Trait:
- Geriatric Depression Scale (GDS)
- UCLA Loneliness Scale (UCLA-L)
- Perceived Stress Scale (PSS)
- Hospital Anxiety and Depression Scale (HADS)

Mindfulness:
- Mindful Attention Awareness Scale (MASS)

Sleep / Circadian:
- Composite Scale of Morningness (CSM)
- Pittsburgh Sleep Quality Index (PSQI)

Menopausal:
- EPICC Menopause Questionnaire

Activity:
- Sitting Time Questionnaire
- Godlin Leisure Time Exercise Questionnaire

Session 3: If the subject is deemed 'cognitively normal' they will be scheduled for the third session – the fitness assessment. Participants will complete a maximal VO_2 test to assess aerobic capacity following a Modified Balke Protocol with an agreed upon speed between participant and lead exercise physiologist. The session will first start by a warm-up session of 5 minutes followed by a blood-pressure reading. Only participants with safe blood pressure readings will be allowed to continue. The participant will walk on a motor-driven treadmill with constant speed and increments of the incline. The intensity is increased in two-minute stages. We will conduct a maximal graded exercise test to determine the peak oxygen capacity for each participant. During this test, we will collect all of the exhaled air of the participant. This is done by having the subject wear a fitted facemask that collects the exhaled air which then travels through a tube to the analyzer. This assessment usually takes about 15 - 30 minutes to complete depending on the starting fitness level of the participant. These procedures follow procedures recommended by the American College of Sports Medicine for graded exercise testing. Medical personnel will be in the immediate vicinity of the exercise test for emergencies. During the exercise test, heart rate is continuously monitored via ECG along with blood pressure readings and Rating of Perceived Exhaustion (RPE) every two minutes. When the subject reaches the endpoint goal of the exercise test (symptom limitation and/or volitional exhaustion), the facemask will be removed and they will undergo a four-minute active cool-down in which they will walk at a slower rate with zero incline grade. After these four minutes the participant will be helped off of the treadmill and undergo a passive cool-down session in which they are seated in a chair. The participant's heart rate and blood pressure will be continuously monitored through the cool-down period every two minutes and the participant will not be allowed to leave until the vital signs return to normal patterns (within a value of 20 mm Hg of systolic and diastolic and 20 beats per minute of heart rate).

Each maximal exercise test will be administered by a trained exercise physiologist and assistant (both referred to as exercise technicians). The assistant should be the staff member who records the required values of each stage and assist with test administration. The assistant should be another exercise physiologist, nurse, or staff with a medical background. All exercise testing staff must complete the IGNITE certification standards. The exercise testing will be used to measure changes in fitness levels after the 12-month intervention. Each testing lab site is expected to have the room set at a comfortable temperature (recommended between 60 – 70 degrees Fahrenheit), have necessary fans in place and have access to water for the participants.

Also at this visit, participants will be fitted with a commercially available physical activity monitoring device (Actigraph Link) around the non-dominant hand wrist that will record objective physical activity data over the duration of

approximately one week. The participants will be provided with detailed instructions regarding wearing of the device as well as the option to remove the device if it becomes problematic. Participants will complete a series of questionnaires at this visit.

Site locations have the option of completing the Senior Fitness Test and Short Physical Performance Battery (SPPB) at this session or during session 5. If completed during session 3, it must be done prior to the VO2 max exercise test.

- Session 4: The fourth session will be an MRI Scan. It is anticipated that the scan will take 1.5 hours to complete. Participants will be scheduled for the MRI scan and standard sequences will be collected that will consist of images of brain structure, cerebral blood flow, and function. To assess brain function, we will use an n-back paradigm that measures working memory and the Relational and Item Specific Encoding Task (RiSE) which is a hippocampal-sensitive task to measure hippocampal function (see descriptions below). Before entering the MRI machine participants will undergo an additional safety screen by the MRI center and will be instructed about the task and be allowed to practice the tasks that they will be performing along with completing a questionnaire. After performing the practice tasks, they will complete the Big Five Trait Taxonomy II (BFI) questionnaire. All data collected during this session will then be transferred to the coordinating center for quality control assessment. During this session, participants will complete two tasks while in the MRI

N-Back has the subject presented with a sequence of stimuli, and the task consists of indicating when the current stimulus matches the one from n steps earlier in the sequence. The load factor n can be adjusted to make the task more or less difficult. Measures working memory.

The Relational and Item Specific Encoding Task (RiSE) requires participants to alternate encoding tasks between blocks rather than individual trials, and uses confidence ratings and a receiver operator characteristics analysis rather than a remember/know procedure to reduce meta-cognitive demands and obtain independent estimates of familiarity versus recollection based retrieval.

MRI Sequences:
- Scout
- MPRAGE
- T2 Hippocampus
- Resting State fMRI
- N-Back
- RISE

- DTI (Diffusion Spectral Image)
- pCASL
- Flair
- T2 myelin mapping

If the research team discovers an incident or abnormality (e.g., tumor) as a part of the MRI scan, it is important for the study subject to be informed (a) that these scans were not designed for clinical purposes and so a clear diagnosis and prognosis cannot be made, but (b) that if an incidental finding or abnormality is found in the scan that we will have a board certified neuroradiologist at the University of Pittsburgh (coordinating center) assess the scan and provide feedback to the Principal Investigator to pass along to the subject. This may require the site Principal Investigator to recommend that the subject inform their personal physician about the abnormality and request follow-up procedures that are outside the purview of the research study.

Session 5: Participants will then be scheduled for a fifth baseline session. The fifth session will include DXA, a blood draw and pulse wave velocity. Since this visit includes a 10-hour fasting blood draw, it will be scheduled in the morning hours. Recommended session flow is Height and Weight (if not using the values collected during VO2), Pulse Wave Velocity, Blood Draw and Hair Sample, Snack and Questionnaires, DXA Scan and Functional testing if not completed during session 3 (SPPB and Sr. Fitness).

BLOOD DRAW:

After blood is collected, the subject will be provided with a light snack and plenty of water before and up to the actual blood draw and to take medications on their normal schedule. It will be promoted to consume water to allow for easier access to veins for the actual blood draw along with maintaining a normal blood pressure in order to prevent dizziness and associated weakness. At each blood draw we will collect approximately 60 cc of blood for a total of 180 cc for each subject during the duration of the study (we will collect blood at baseline, 6-months, and 12-months). Blood sampling will be postponed in subjects experiencing an acute infection (e.g., respiratory or flu-like symptoms).

HAIR SAMPLE TO MEASURE CORTISOL LEVELS:

The hair sample collection is a new method used to measure the amount of the stress hormone cortisol found in the body. Cortisol is implicated in the

development of many common conditions and this new technique could allow us to study its role better. Hair must be 1cm (.40 inches) in length and it will be cut from the back of the scalp (not pulled out). Approximately the diameter of pencil is to be cut (approximately 40 strands of hair).

PULSE-WAVE VELOCITY (PWV):

PWV will be assessed using ultrasound. Following 10 minutes of supine resting, the participant will be asked questions about when they last had caffeine, nicotine, and food, and exercise. Technicians will obtain two supine blood pressure measurements using an automated blood pressure device on the right arm (DINAMAP CARESCAPE V100, GE Healthcare, Milwaukee, WI) both immediately prior to and immediately following PWV measurement as described below. To measure PWV, the technician will palpate the carotid and femoral arteries on the right side of the patient to locate testing sites. The technician will measure the distance from the carotid artery to the suprasternal notch and the distance from the suprasternal notch to the femoral artery using a tape measure. The aortic distance is calculated as the latter (suprasternal notch to femoral) minus the former (suprasternal notch to carotid) in meters. Piezoelectric (or pressure measuring) sensors are applied by the technician to the carotid and femoral arteries to measure the time differential in the arrival of pulse waves (measured as the foot of each wave) created by the heartbeat as they arrive at the carotid and femoral testing sites, respectively. Data are collected in triplicate so the results can be averaged, reducing measurement variability.

BODY COMPOSITION (10 min):

Body composition will be assessed via dual-energy x-ray absorptiometry (DXA). Full-body DXA scans provide data for fat mass, fat-free mass, % body fat, and bone mineral density. Subjects will be clothed in a lightweight hospital gown and will be instructed to remove all jewelry, hairpins, etc. that would potentially affect the accuracy of this measurement. The scanner will be calibrated each day according to the guidelines specified by the manufacturer.

Session 6: The final session will be completed only at the baseline time point and it is expected to be approximately a 2.5-hour PET session. The PET scan will not be completed at Northeastern University. Preparation of the subject for the PET scan (venous catheterization) and after the scan for return home will be performed by an RN who has over 5 years of experience working on Amyloid PET scans. Participants will be asked to fast for at least one hour prior to the scheduled scan time. They will be asked to avoid all caffeinated beverages (coffee, tea, soft drinks, etc.) on the day of the PET scanning. They will be asked to avoid all alcoholic beverages for 48 hours before the PET scanning. They may take prescribed medications at their usual times. A trained nurse will place a

venous catheter in a vein in the arm or hand so that the radio-tracer can be injected during the study.

With holes for the eyes, ears, and mouth placed over the subject's face, brain Amyloid will be measured using PET with an FDA-approved radioactive substance called Neuraceq (Florbetaben F18). A standard dose of the Neuraceq [8.0 mCi (+/- 10%)] will be injected in a vein in the arm and subjects will sit quietly in a chair for 50 minutes. Following this, subjects will be positioned on the scanner bed and a 10 - 15-minute transmission scan will be performed using either rotating Ge-68/Ga-68 rods or a low dose CT scan of the head followed by the scanner taking pictures for 20 minutes. During the scans, subjects must hold their head as still as possible. After the scans are completed, subjects will be asked to drink fluids and empty their bladder. No subject will receive more than 1 administration (8.0mCi) of Neuraceq in a given study year.

1.7.4 POST RANDOMIZATION TASKS:

The self-efficacy questionnaire will be completed during the third week of the intervention. At this time, the diet history (NCI) will be given to study participants to take home to complete.

Actigraph Link will be worn for one week every other month for each study participant. The coordinating center will maintain a calendar for each study site to when study participants are to wear the device.

1.7.5 TIME POINT 2 (6 months):

At 6-months into the exercise sessions, the participants will complete two cognitive assessments similar to that of the baseline visit. The tasks will be the same as those described above but will not include the WTAR, the Health History questionnaire, the ECOG, the MOCA, and the CIRS. However, no adjudication will be done on any of the cognitive data collected at this time point. This time point also includes a blood draw to collect the same biomarkers as the Time point 1 blood draw.

1.7.6 TIME POINT 3 (12 months – post intervention):

At the 12-month session, participants will complete the same first five sessions as done during baseline except for the last session (Session 6 – PET scan). These sessions will be conducted as described above. The only changes will be that the cognitive sessions will not include the administration of the WTAR, the CIRS, the ECOG, and the Health History.

1.8 MRI READING FOR INCIDENTAL FINDINGS:

Across our primary investigators (Erickson, Burns, Kramer, Hillman, McAuley) we have conducted well more than 2000 MRI scans in cognitively normal and healthy older adults. Based on these data we expect that about 5% of participants will show clinical abnormalities. Nonetheless, we recognize that there should be significant care taken for these participants with unanticipated findings and we have documented a process described below for handing these cases. It should also be recognized that our MRI sequences are designed for research purposes and not designed for assessing clinical abnormalities. Therefore, even the most thorough read of an abnormality would require the participant to follow-up with their PCP or hospital physician for further testing.

To reduce the potential concern about undetected abnormalities in the MRI data, we will consult with a neuroradiologist at the University of Pittsburgh (Dr. Mettenburg). All images for a study participant will be sent to Dr. Mettenburg in a weekly batch upload of all recently completed MRI scans. The original dicoms will be sent to the IGNITE folder in the PACS system of the University of Pittsburgh Medical Center. All images will retain confidentiality by having no identifiable information. It is expected that Dr. Mettenburg will complete the MRI read within 7 days of being loaded onto the PACS system. After examining the MRI scans, Dr. Mettenburg will complete MRI Read Form directly in REDCap. The imaging director at the coordinating center will have completed box 1 on the document regarding the time of image transfers. This allows for tracking in REDCap of images sent in each batch to be read by the Neuroradiologist. Dr. Mettenburg will check the level of finding in section 2 of the MRI read document. The levels of findings are categorized into one of four categories:

1. **NO CONSEQUENCE**: Nothing is communicated with participant as images are usual or expected.
2. **NO FOLLOW-UP RECOMMENDED:** Study participant is informed that something was seen but it does not warrant a follow-up with PCP.
3. **FOLLOW-UP RECOMMENDED:** A finding has been verified and it is recommended that the study participant follow-up with their PCP.
4. **EMERGENT:** Participant should be notified immediately by study PI and seek immediate medical care.

If an incidental finding has been verified (options 2 – 4), Dr. Mettenburg will complete section 3 of the document that will include details of findings. It is then the responsibility of the site PI to follow up as appropriate with clinical referrals and report the finding to IRB and study DSMB. For any recommended follow-up or emergent findings, these cases will be discussed by the executive committee members for a decision if study participant is eligible to continue in the trial. If required, the site PI will complete section 4 of the MRI read form. Section 4 would include any notes on communication with study participant along with discussion regarding eligibility within executive committee. The participant will then work with their PCP or hospital for these follow-up clinical appointments at their own time and cost.

1.9 <u>RANDOMIZATION</u>:

After the completion of the measures and sessions described above the participants will be randomly assigned to one of three groups. These include (a) stretching and toning control condition, (b) an exercise condition that will consist of 150 minutes per week of moderate intensity exercise, or (c) exercise condition that will consist of 225 minutes per week of exercise. We will use a computer randomization protocol that will incorporate a verification that all session visits have been completed and data entered into the database. We will use a minimization algorithm with equal allocation to one of the three groups. The use of the minimization strategy will ensure treatment balance on the two factors of age at study entry (=<72, >72) and gender. Once randomized, an assigned staff member will contact the subject via phone with the group information. On this contact, the staff member will give information such as date, day, time, and location of the subject's first intervention session. If a couple (husband and wife) pass screening, they will be randomized as one in the same group.

1.9.1 <u>Targeted Enrollment Table for Pittsburgh:</u>

TARGETED/PLANNED ENROLLMENT: Number of Subjects			
**Ethnic Category**	**Females**	**Males**	**Total**
Hispanic or Latino	3	3	6
Not Hispanic or Latino	125	82	207
**Ethnic Category: Total of All Subjects ***	128	85	213
**Racial Categories**			
American Indian/Alaska Native	1	0	1
Asian	5	3	8
Native Hawaiian or Other Pacific Islander	0	0	0
Black or African American	33	22	55
White	89	64	153
**Racial Categories: Total of All Subjects ***	128	85	213

* The "Ethnic Category: Total of All Subjects" must be equal to the "Racial Categories: Total of All Subjects."

1.9.2 Targeted Enrollment Table for Kansas City:

TARGETED/PLANNED ENROLLMENT: Number of Subjects			
**Ethnic Category**	**Females**	**Males**	**Total**
Hispanic or Latino	3	3	6
Not Hispanic or Latino	125	82	207
**Ethnic Category: Total of All Subjects ***	128	85	213
**Racial Categories**			
American Indian/Alaska Native	1	0	1
Asian	2	1	3
Native Hawaiian or Other Pacific Islander	0	0	0
Black or African American	14	9	23
White	111	75	186
**Racial Categories: Total of All Subjects ***	128	85	213

* The "Ethnic Category: Total of All Subjects" must be equal to the "Racial Categories: Total of All Subjects."

1.9.3 Targeted Enrollment Table for Boston:

TARGETED/PLANNED ENROLLMENT: Number of Subjects			
**Ethnic Category**	**Females**	**Males**	**Total**
Hispanic or Latino	18	12	30
Not Hispanic or Latino	110	73	183
**Ethnic Category: Total of All Subjects ***	128	85	213
**Racial Categories**			
American Indian/Alaska Native	1	0	1
Asian	9	6	15
Native Hawaiian or Other Pacific Islander	0	0	0
Black or African American	32	21	53
White	86	58	144
**Racial Categories: Total of All Subjects ***	128	85	213

* The "Ethnic Category: Total of All Subjects" must be equal to the "Racial Categories: Total of All Subjects."

1.10 EXERCISE INTERVENTION GROUPS:

1.10.1 AEROBIC EXERCISE GROUPS: Participants in the two aerobic exercise conditions will begin by walking for 15-20 minutes during the first week of the

program and gradually increase their walking duration by five minutes each subsequent week until they reach 30-45 minutes of walking depending on the group (see more below). All walking sessions start and end with 5-10 minutes of stretching for the purpose of warming up and cooling down. Levels of exercise intensity will be prescribed based upon maximal responses during the initial graded exercise treadmill test. As the participants will be low active at baseline, the prescribed intensity will be 50–60% of the maximum heart rate reserve for weeks one to six and 60–75% for the remainder of the program. Participants in the walking conditions will wear heart rate monitors and be encouraged to walk within their target heart rate zone. Heart rate intensity will be measured by Polar A360 devices worn around the wrist like a watch. Certified exercise instructors will closely monitor attendance, intensity, frequency, and safety. On-site exercise sessions will occur three days/week for the duration of the intervention and at home for two other days of the week. Compliance to home-based exercise will be monitored by exercise diaries. For the group receiving 150 minutes of exercise, they will be encouraged to briskly walk for up to 30 minutes in the facility and at home for two other days of the week at a similar intensity. For the group receiving 225 minutes/week a walking duration of 45 minutes will be achieved by the sixth week of the program. Participants will be instructed to exercise at home for 45 minutes for two other days of the week at a similar intensity.

1.10.2 STRETCH AND TONE GROUP:

The control group will focus on improving balance, flexibility, and strength. Resistance bands, balance disks, yoga blocks, and exercise mats will be used to engage in non-cardiorespiratory activities. This goal for this group is 150 minutes of exercise per week (3 supervised sessions and 2 home based sessions each at 30 minutes). These are progressive in nature and include modifications to accommodate injuries, physical difficulties, etc. A new set of similar activities will be introduced every week. There are four styles of classes that will be rotated through each week, they include Yoga / Mobility, Total Body Fusion, Pilates / Balance, and Stretch / Tone. Participants in this group also will have logs completed at each session and receive monthly feedback forms. They will be monitored continuously through class so intensity does not reach heart rates above 100 beats per minute. This corresponds to 9 - 10 on the Borg RPE scale. Adherence is tracked and study participants are encouraged to attend as many classes as possible of the required on-site exercise sessions (3 classes per week). Each class is expected to last 30 minutes in length with 5 minutes each of warm up and cool down periods. Participants will complete home logs to monitor sessions completed at home.

1.10.3 EXERCISE COMPLIANCE:

Participants in the study will be completing part of the exercise under supervised conditions and part of the exercise under unsupervised conditions (e.g., home exercise). Monitoring compliance during the supervised exercise is relatively straightforward because we will have trainers monitoring attendance, duration of exercise, perceived exertion, mode of activity, and heart rate intensity throughout the period. However, compliance during the unsupervised sessions is inherently more challenging. In this study, we have proposed a two-faceted approach for monitoring compliance during the unsupervised periods. First, within the first few weeks of the intervention, our staff will train participants on the Ratings of Perceived Exertion (RPE) Scale and participants will be instructed to keep weekly diaries of the times, durations, and RPE intensity during the unsupervised exercise that they engage in. These diaries will then be collected on a weekly basis and entered into our database (REDcap). These diaries are a natural component of training since training involves the identification of barriers and approaches to overcoming non-compliance while keeping individuals engaged and continuing to return for the sessions.

In addition to the self-reported diaries of unsupervised exercise, we will have participants wear an Actigraph accelerometer for 1 week every other month of the study for 12 months (~7 weeks of accelerometer data). We will be able to use these data as objective confirmation that when participants are recording their exercise in the diaries they are doing the activity at the prescribed intensity and for the prescribed duration. These assessments are important for providing an objective assessment of compliance during the unsupervised exercise periods.

Based on the above, we have operationally defined compliance to the intervention as those participants meeting the prescription of either an average of 225 minutes per week of moderate intensity exercise (Group 1), 150 minutes per week of moderate intensity exercise (Group 2), or 150 minutes per week of light intensity stretching (Group 3). Compliance to these prescriptions will be based on the information gathered from the weekly diaries, the supervised exercise sessions, and the accelerometer recordings. We will record on a weekly basis for each participant whether they were compliant or not.

Our analysis plan has proposed an intent-to-treat analysis where all participants, regardless of whether they complete all sessions, will be invited to return for follow-up assessments. However, there will likely be some participants that refuse to return for follow-up assessments or miss a mid-point assessment (cognitive and blood will be collected at the 6 month period). “Lost to follow up” will be defined here as missing data that is contributing to the primary outcome (executive function composite). The reason will be recorded (i.e., missed visit due to traveling, illness). These criteria and documentation will also be applied for any discontinued subjects with the reasons for discontinuation being recorded (i.e., moving out of state).

Regarding the analytical approach, we propose to use all available data in the planned analysis for testing our primary outcome. Since we are examining changes over time we will be able to conduct the analysis as long as there is at least one follow-up assessment (either at the 6-month time point or the 12-month time point). We will assess the missingness of data consistent with the assumptions of the modeling approach described above. That is, we will examine the randomness of missing data and determine if the data is missing completely at random (MCAR) or missing at random (MAR) and use maximum likelihood estimation for computation of unbiased parameters estimates and standard errors. We will not conduct multiple imputation to correct for missing data.

1.11 PARTICIPANT SAFETY:

The safety of study participants will be monitored throughout the study. Study participants will undergo an extensive screening prior to entering the trial to determine that it is safe for them to participate in the intervention. Safety is continuously monitored during the intervention via three supervised sessions per week. We are aware of the potential for serious adverse events to occur with any type of exercise training. As such, the trial will take measures to reduce the risk of an adverse event occurring. All individuals involved in human subject's research, regardless of whether exercise training is involved, are trained and certified in CPR and First Aid. Also, we have budgeted funds to hire medical personnel to oversee all exercise stress testing to ensure the safety of the study participants. Included in the exercise stress testing room is an automated external defibrillator (AED), should it be deemed necessary. Lastly, protocols are currently in place to respond to any adverse events that include contacting emergency response personnel and facilitating their arrival to the correct rooms within the buildings at each university.

Each IGNITE study investigator has responsibility for the safety of study participants at their site. The Data Safety Monitoring Board (DSMB), is responsible for monitoring study data for evidence of adverse effects attributed to the intervention. The DSMB also has the authority to recommend changes, stopping the trial at any time, or pausing the trial until problems are resolved. All AEs experienced by the participant during the study (Consent signing until end of study outcome collection) are to be reported. The IGNITE trial will track the occurrence of:

- Serious Adverse Events (SAE)
- Unexpected events
- Unfavorable medical events that occur during intervention sessions

The trial has created two forms for adverse event reporting. These documents have been created based on the belief that trainers and staff should not be responsible for determining whether the event is related to the intervention or whether/how it should be reported to the IRB and DSMB. Only the site PI (and if necessary the executive committee) will determine if the event is (a) expected or unexpected, and (b) related to the intervention. Given the frequency of the interactions between subjects and trainers it will be important for trainers and staff to ask if the participant has had any illnesses, injuries, or medication changes since their last supervised exercise session. Based on responses to these questions, regardless of seriousness or relatedness, the trainer will complete a form documenting an adverse event. We have created a brief form for trainers (IGNITE Adverse Event Form for Trainers) for this purpose. Trainers or staff would first document the event on this form. This form allows trainers to circle the reported event (or provide an event not listed on the form). This completed document would be sent to the study site coordinator who would then speak to the trainer and/or participant to find out more information about the event. The site coordinator would then complete the IGNITE Event Evaluation Form based on this communication. This form has several sections with boxes available for documenting the reported severity (mild, moderate, severe), the chronicity (single occurrence, intermittent, persistent), whether the event was resolved or not, and whether it was related to the intervention and expected or unexpected. In particular, these forms are intended to clarify for the investigators, the IRB, and the DSMB, whether the adverse event was serious, whether the adverse event was related to the intervention, and whether it was expected or unexpected. An example of an expected adverse event from the exercise intervention would be muscle soreness which would be listed as a known risk in the IRB. An unexpected adverse event related to the intervention is if someone falls off a treadmill and injures an ankle. The site PI will oversee the determination of these events and if there is any question about whether the event was related to the intervention, its seriousness or expectedness, the event will be discussed on the weekly executive committee call. For DSMB purposes, all adverse events that are reported will be recorded and separated based on whether the event was related to the intervention, expectedness, its seriousness, severity, chronicity, and resolution.

1.12 DATA MANAGEMENT:

We appreciate the challenges associated with the organization, execution, management, and analysis of a study of this kind and recognize the importance of frequent communication between staff, students, analysts, and investigators. Protection of subject privacy and safety, meticulous QC, and prudent organization is the bedrock for success of a study like this, which will require a sizeable team of staff and students. For this study, Dr. Erickson will maintain overall responsibility for DM and QC, but we have constructed a DM plan that distributes database and analysis workload across labs

and specialization. Specifically:

- Dr. Erickson will lead DM, organization, and QC of the cognitive, neuroimaging, questionnaires, fitness, physical activity, DXA, outcomes as well as demographic information, adverse events, standardization of assessments, compliance, and adherence of the intervention protocol.
- Dr. Marsland will lead the effort for the blood assays
- Dr. Kamboh for genetics
- Drs. Kang and Tseng (biostatisticians) will monitor database integrity for missing data, errors, outliers, and distributions.

IT staff will control permissions to the database to ensure proper access and DM by staff, students, and investigators. We will store and link all behavioral and assay data on a HIPPA secure cloud-based server (REDcap) and will use the NIH-recommended and supported XNAT application for securely storing and accessing imaging data from multi-site studies. Both REDcap and XNAT allow investigators at all sites to access data and coordinate analyses.

Data entry to the REDcap database will be completed at the coordinating center at the University of Pittsburgh. The data forms in REDcap are similar to the actual paper version of the forms that will be completed by participants and staff. All participant data will be stored and archived on a secure server at the Coordinating Center at the University of Pittsburgh. The server is set for safe mode that allows two copies of each data to be saved on the server. The data server at the coordinating center has a hard drive array, which is composed of a few hard drive disks. If one or two of the disks fail, the rest of the disks will back up the data of the failed disks automatically. By having the data base on REDCap, all data is secure is case of a natural disaster in which the server would be completely undamaged.

<u>Missed Visits:</u> All outcome collection must be reported and stored in the database. Therefore, if an outcome visit is missed or not collected, a missed visit form must be completed in REDCap. Once a missed visit form is completed, the data management team at the coordinating center will indicate in the database that the outcome data for the specific measure is missed to follow up. All missing data in the database will require additional documentation regarding whether the data are "lost to follow up" or has been collected but there was a problem with transferring the data to the coordinating center.

The missed visit form has three sections that must be completed:

- Time point of the missed visit
- Outcome that was missed
- Reason the outcome was missed

If a study participant misses multiple outcome measures during the same time point, one missed visit form can be completed if the reason missed is the same for each measure. Thus, staff do not have to complete a new form for each missed outcome for the same time point. For example, at midpoint, if the study participant missed cognitive session #1, cognitive session #2, and blood draw because the participant moved out of the area, this information could be completed on one missed visit form. If this study participant would also miss post intervention visits, a new form must be generated since this is a different time point.

Cognitive analysis DM plan: We will conduct confirmatory factor analyses to determine whether the cognitive results load onto domains found in prior studies. These cognitive domains will be used for all higher-level analyses (described below) as to (a) reduce inflated error rates from multiple cognitive tests, and (b) allow interpretations from the study to be applied to cognitive domains (e.g., executive function) rather than individual tests (e.g., Stroop).

Imaging data analysis and DM plan: We will first analyze each imaging outcome (e.g., volume) separately from every other imaging outcome (e.g., fMRI). But, once voxel-wise associations related to the interventions are established, we could test, using hierarchical regression, the relative significance of each imaging measure to explain improvements in cognitive function with the intervention. There are also other analyses that would allow us to integrate the multi-modal imaging outcomes. For example, we could utilize machine-learning approaches to predict, based on baseline imaging measures, adherence and/or responsiveness to the PA intervention (a "brain-as-predictor" approach). We will also have the capability to answer multi-modal imaging questions, allowing us to pinpoint the (as of yet) elusive physiological underpinnings of exercise-induced brain changes (e.g., do changes in myelin mediate volumetric changes in the hippocampus).

Biomediator DM plan: We will test for co-linearity between the cardiovascular and metabolic factors and examine fit for a latent structure when testing mediation. Similar approaches will be used to collapse across analytes (i.e., inflammatory cytokines) so as to reduce error associated with multiple testing of correlated measures. When significant effects are detected in higher-level analyses (see below) we will conduct secondary analyses to determine which variable(s) (e.g., IL-6) are contributing to the association.

1.13 QUALITY CONTROL:

The multi-site nature of this RCT demands rigorous QC to ensure consistency of the intervention procedures and protocols across sites. Staff at all three sites will convene early in the first year to standardize protocols. We will hold monthly conference calls with staff to ensure continuation of all protocols. Throughout the study

period, Dr. Gothe, who will be external to all sites, will conduct annual QC checks. She will evaluate the implementation of the intervention and exercise prescription by the instructors and exercise leaders, adherence to the intervention protocols, training of the personnel, and consistent collection and monitoring of PA data via weekly logs. The competency and QC for the instructors and exercise leaders will be monitored by direct observation during supervised exercise sessions. Written and verbal feedback of QC will be provided to the PIs and exercise leaders. Reports of QC will be a standard agenda item for the annual meeting of investigators and the Data Safety and Monitoring Board (DSMB) meetings. During the course of the study we anticipate a turnaround of staff, graduate and undergraduate assistants, and these QC visits will ensure compliance and uniformity of study protocols.

1.13.1 COGNITIVE AND MRI QUALITY CONTROL:

We recognize the importance and challenge of maintaining QC of cognitive and neuroimaging data in a multi-site study. To maintain QC of the cognitive data, administrators at all sites will receive QC training in Year 01 to administer the tests. These certification sessions will be conducted annually for maintenance of QC. Dr. Erickson's team will merge raw data across sites (concomitant with its collection) and monitor site-specific differences that may reflect a change in QC at one of the sites. Staff in Dr. Erickson's laboratory will be responsible for scoring all of the cognitive data and sharing it with Drs. Butters and Becker for adjudication. If site-specific variation is identified, investigators at all sites will address the issue before continued data collection at that site.

To maintain QC of the neuroimaging data, co-I Sutton will establish identical MR sequences at all sites. Each site maintains Siemens 3T machines (Pitt and Northeastern have Prisma scanner while Kansas has Skyra). Each site will have weekly QC checks using phantoms and QC will be monitored according to BIRN criteria, established by other multi-site imaging studies. Before the start of the intervention, several investigators will be scanned at each site to ensure equivalent contrast and image quality for each sequence. All imaging data will be uploaded to XNAT and will be examined by Dr. Erickson's laboratory to assess QC, proper sequences, and site- specific effects.

1.14 STUDY LIMITATIONS:

There may be questions about whether our doses will be sufficient for detecting effects on brain or cognitive outcomes. We selected these doses based on widely prescribed recommendations and preliminary data, but it is possible that less activity would be sufficient or greater amounts of activity would prove better. There may also be concerns about power related to genetic associations. This is a fair critique given the usually small effect sizes and skewed distributions associated with risk alleles. We will focus on SNPs in preselected pathways described above to alleviate this problem. We

will acknowledge in any manuscripts the challenges in interpreting statistical interactions with risk alleles. There may also be concerns about practice effects for our cognitive battery as it will be employed 3 times. To address this concern, we have selected tests that have alternative versions and will focus our analyses on Group x Time effects as both the intervention groups and the control group will encounter the cognitive tasks an equivalent amount of time. In addition, we plan to use time as a fixed effect to control for any systematic differences between groups (i.e., practice effects) and then compare group differences after controlling for these learning effects. We have also elected to not follow participants after intervention completion, but we expect to submit ancillary proposals to continue to follow participants to explore long-term changes in cognitive, brain, or biomarker outcomes.

1.15 RESOURCE SHARING:

1.15.1 DISSEMINATION PLAN:

We will develop an extensive dissemination plan and will publicize the results of the study to practicing clinicians, policy makers, research study participants and the general public by utilizing diverse strategies outlined below. Widespread dissemination will occur during the year following trial completion and publication with the main results and secondary and ancillary results by employing the following techniques: (a) media coverage through press releases and interviews targeted to local and national newspapers, television and radio outlets; (b) production of a research summary document and "facts sheet" targeted to the general public which clearly and concisely summarizes the key conclusions of the trial; (c) production of professionally designed flyers, posters, brochures, and research briefs targeted to broad audiences; (d) use of concise policy briefs to advocate for legislative and policy change at local, state, and national levels, which focus on how new evidence has implications for a particular policy; (e) study newsletters targeted to study participants; (f) distribution of dissemination materials to community agencies, professional societies and health-related websites and list-serves; (g) hosting and attending seminars, conferences, community forums and health fairs; (h) mailing personal thank you letters to research study participants; (i) developing an IGNITE study website to share news and research findings to study participants and the public.

1.15.2 DATA SHARING:

We are familiar with the NIH policies regarding data sharing and will comply with local, state, and federal laws, such as the Privacy Rule, a Federal regulation under the Health Insurance Portability and Accountability Act (HIPAA). We will follow the NIH data sharing guide and provide a data sharing plan to be reviewed and approved by the relevant NIA PO. We recommend that data sharing be accomplished using mixed modes, each providing a different level of access. This

will include data enclave (controlled, secure environment in which eligible researchers can perform analyses using data resources), data archive (place where machine-readable data are acquired, manipulated, documented, and distributed), researcher's efforts (investigator responds to data requests), and publishing articles in scientific publications. We propose to use **REDcap** (Research Electronic Data Capture) as a data enclave for investigators that may request access to certain data. REDcap is a secure web application for building and managing surveys and databases. It allows a streamlined process for rapidly creating and organizing databases and allows for automated exporting of data to Excel and common statistical packages (SPSS, SAS, Stata, R) as well as a built-in project calendar, a scheduling module, ad hoc reporting tools, and other features. In addition, we propose to use **XNAT**, an informatics platform for imaging research. XNAT is an open-source imaging informatics software platform dedicated to helping perform imaging-based research. XNAT's core functions include managing, importing, archiving, processing, and securely distributing imaging data across multiple sites. This tool includes full DICOM image integration and anonymization, secure access and permission controls, and other features. XNAT is being employed and recommend by the NIH-supported BIRN (Biomedical Informatics Research Network) for multi-site neuroimaging studies as a rapid and secure method for data sharing and quality control.

1.15.3 DATA SHARING AGREEMENT:

The data-sharing agreement provides for: (1) a commitment to using the data only for research purposes and not to identify any individual human participant; (2) a commitment to securing the data using appropriate computer technology; and (3) a commitment to destroying or returning the data after analyses are completed. We will make the data available to outside investigators after publication of the primary aims and at completion of quality control assessments and data organization. We expect that the data from this study will result in significant interest from internal and external investigators for secondary data analysis and ancillary project proposals, so we will create an oversight committee made up of Principal and Co-Investigators for monitoring, approving, and disseminating the data along with monitoring data analysis plans and development of manuscripts. This is important for eliminating redundant analyses, results, or manuscripts. This Presentation and Paper (P&P) committee will be responsible for ensuring that (a) when an investigator requests access to data that the particular question being proposed is not already being addressed by another investigator, (b) that the proposed analytic plan and hypotheses are sound, and (c) that the investigator proposing the analysis has the resources and expertise to conduct the proposed analysis. Protecting the rights and privacy of human subjects will be our first priority. The final datasets will be de-identified prior to release for sharing.

1.16 REFERENCES:

1. Association As. Alzheimer's Disease Facts and Figures. *Alzheimer's and Dementia.* 2010;6.

2. Bureau UC. American Community Survey. 2010.

3. Petersen RC, Caracciolo B, Brayne C, Gauthier S, Jelic V, Fratiglioni L. Mild cognitive impairment: a concept in evolution. *Journal of internal medicine.* 2014;275(3):214<228.

4. Barnes PM. *National Health Statistics Reports.* 2008.

5. Albert MS. Changes in cognition. *Neurobiology of aging.* 2011;32 Suppl 1:S58<63.

6. Young J, Angevaren M, Rusted J, Tabet N. Aerobic exercise to improve cognitive function in older people without known cognitive impairment. *The Cochrane database of systematic reviews.* 2015;4:CD005381.

7. Angevaren M, Aufdemkampe G, Verhaar HJ, Aleman A, Vanhees L. Physical activity and enhanced fitness to improve cognitive function in older people without known cognitive impairment. *The Cochrane database of systematic reviews.* 2008(3):CD005381.

8. Colcombe S, Kramer AF. Fitness effects on the cognitive function of older adults: a meta<analytic study. *Psychological science.* 2003;14(2):125<130.

9. Hindin SB, Zelinski EM. Extended practice and aerobic exercise interventions benefit untrained cognitive outcomes in older adults: a meta<analysis. *Journal of the American Geriatrics Society.* 2012;60(1):136<141.

10. Smith PJ, Blumenthal JA, Hoffman BM, et al. Aerobic exercise and neurocognitive performance: a meta<analytic review of randomized controlled trials. *Psychosomatic medicine.* 2010;72(3):239< 252.

11. Blazer DG, Yaffe K, Karlawish J. Cognitive Aging: A Report From the Institute of Medicine. *JAMA: the journal of the American Medical Association.* 2015.

12. Fjell AM, Westlye LT, Grydeland H, et al. Critical ages in the life course of the adult brain: nonlinear subcortical aging. *Neurobiology of aging.* 2013;34(10):2239<2247.

13. Lovden M, Schmiedek F, Kennedy KM, Rodrigue KM, Lindenberger U, Raz N. Does variability in cognitive performance correlate with frontal brain volume?

NeuroImage. 2013; 64:209<215.

14. Raz N, Ghisletta P, Rodrigue KM, Kennedy KM, Lindenberger U. Trajectories of brain aging in middle<aged and older adults: regional and individual differences. *NeuroImage.* 2010;51(2):501< 511.

15. Rodrigue KM, Haacke EM, Raz N. Differential effects of age and history of hypertension on regional brain volumes and iron. *NeuroImage.* 2011;54(2):750<759.

16. Kennedy KM, Erickson KI, Rodrigue KM, et al. Age<related differences in regional brain volumes: a comparison of optimized voxel<based morphometry to manual volumetry. *Neurobiology of aging.* 2009;30(10):1657<1676.

17. Raz N, Rodrigue KM, Haacke EM. Brain aging and its modifiers: insights from in vivo neuromorphometry and susceptibility weighted imaging. *Annals of the New York Academy of Sciences.* 2007; 1097:84<93.

18. Raz N, Rodrigue KM. Differential aging of the brain: patterns, cognitive correlates and modifiers. *Neuroscience and biobehavioral reviews.* 2006;30(6):730<748.

19. Raz N, Lindenberger U, Rodrigue KM, et al. Regional brain changes in aging healthy adults: general trends, individual differences and modifiers. *Cerebral cortex.* 2005;15(11):1676<1689.

20. Brown BM, Peiffer JJ, Martins RN. Multiple effects of physical activity on molecular and cognitive signs of brain aging: can exercise slow neurodegeneration and delay Alzheimer's disease? *Molecular psychiatry.* 2013;18(8):864<874.

21. Erickson KI, Gildengers AG, Butters MA. Physical activity and brain plasticity in late adulthood. *Dialogues in clinical neuroscience.* 2013;15(1):99<108.

22. Gomez<Pinilla F, Hillman C. The influence of exercise on cognitive abilities. *Comprehensive Physiology.* 2013;3(1):403<428.

23. Hillman CH, Erickson KI, Kramer AF. Be smart, exercise your heart: exercise effects on brain and cognition. *Nature reviews. Neuroscience.* 2008;9(1):58<65.

24. Voss MW, Vivar C, Kramer AF, van Praag H. Bridging animal and human models of exercise< induced brain plasticity. *Trends in cognitive sciences.* 2013;17(10):525<544.

25. Barnes DE, Yaffe K. The projected effect of risk factor reduction on Alzheimer's disease prevalence. *Lancet neurology.* 2011;10(9):819<828.

26. Sofi F, Valecchi D, Bacci D, et al. Physical activity and risk of cognitive decline: a meta<analysis of prospective studies. *Journal of internal medicine.* 2011;269(1):107<117.

27. Etnier JL, Nowell PM, Landers DM, Sibley BA. A meta<regression to examine the relationship between aerobic fitness and cognitive performance. *Brain research reviews.* 2006;52(1):119<130.

28. Dustman RE, Ruhling RO, Russell EM, et al. Aerobic exercise training and improved neuropsychological function of older individuals. *Neurobiology of aging.* 1984;5(1):35<42.

29. Kramer AF, Hahn S, Cohen NJ, et al. Ageing, fitness and neurocognitive function. *Nature.* 1999;400(6743):418<419.

30. Lautenschlager NT, Cox KL, Flicker L, et al. Effect of physical activity on cognitive function in older adults at risk for Alzheimer disease: a randomized trial. *JAMA : the journal of the American Medical Association.* 2008;300(9):1027<1037.

31. Erickson KI, Leckie RL, Weinstein AM. Physical activity, fitness, and gray matter volume. *Neurobiology of aging.* 2014;35 Suppl 2:S20<28.

32. Erickson KI, Voss MW, Prakash RS, et al. Exercise training increases size of hippocampus and improves memory. *Proceedings of the National Academy of Sciences of the United States of America.* 2011;108(7):3017<3022.

33. Niemann C, Godde B, Voelcker<Rehage C. Not only cardiovascular, but also coordinative exercise increases hippocampal volume in older adults. *Frontiers in aging neuroscience.* 2014;6:170.

34. ten Brinke LF, Bolandzadeh N, Nagamatsu LS, et al. Aerobic exercise increases hippocampal volume in older women with probable mild cognitive impairment: a 6<month randomised controlled trial. *British journal of sports medicine.* 2015;49(4):248<254.

35. Colcombe SJ, Erickson KI, Scalf PE, et al. Aerobic exercise training increases brain volume in aging humans. *The journals of gerontology. Series A, Biological sciences and medical sciences.* 2006;61(11):1166<1170.

36. Colcombe SJ, Kramer AF, Erickson KI, et al. Cardiovascular fitness, cortical plasticity, and aging. *Proceedings of the National Academy of Sciences of the United States of America.* 2004;101(9):3316< 3321.

37. Smith JC, Nielson KA, Antuono P, et al. Semantic memory functional MRI and cognitive function after exercise intervention in mild cognitive impairment. *Journal*

of Alzheimer's disease : JAD. 2013;37(1):197<215.

38. Suzuki T, Shimada H, Makizako H, et al. A randomized controlled trial of multicomponent exercise in older adults with mild cognitive impairment. *PloS one.* 2013;8(4):e61483.

39. Voss MW, Prakash RS, Erickson KI, et al. Plasticity of brain networks in a randomized intervention trial of exercise training in older adults. *Frontiers in aging neuroscience.* 2010;2.

40. Burdette JH, Laurienti PJ, Espeland MA, et al. Using network science to evaluate exercise< associated brain changes in older adults. *Frontiers in aging neuroscience.* 2010; 2:23.

41. Barnes DE, Santos<Modesitt W, Poelke G, et al. The Mental Activity and eXercise (MAX) trial: a randomized controlled trial to enhance cognitive function in older adults. *JAMA internal medicine.* 2013;173(9):797<804.

42. Blumenthal JA, Emery CF, Madden DJ, et al. Long<term effects of exercise on psychological functioning in older men and women. *Journal of gerontology.* 1991;46(6): P352<361.

43. Sink KM, Espeland MA, Castro CM, et al. Effect of a 24<Month Physical Activity Intervention vs Health Education on Cognitive Outcomes in Sedentary Older Adults: The LIFE Randomized Trial. *JAMA: the journal of the American Medical Association.* 2015;314(8):781<790.

44. Forbes D, Thiessen EJ, Blake CM, Forbes SC, Forbes S. Exercise programs for people with dementia. *The Cochrane database of systematic reviews.* 2013;12:CD006489.

45. Gates N, Fiatarone Singh MA, Sachdev PS, Valenzuela M. The effect of exercise training on cognitive function in older adults with mild cognitive impairment: a meta<analysis of randomized controlled trials. *The American journal of geriatric psychiatry: official journal of the American Association for Geriatric Psychiatry.* 2013;21(11):1086<1097.

46. Colcombe SJ, Erickson KI, Raz N, et al. Aerobic fitness reduces brain tissue loss in aging humans. *The journals of gerontology. Series A, Biological sciences and medical sciences.* 2003;58(2):176<180.

47. Rosano C, Venkatraman VK, Guralnik J, et al. Psychomotor speed and functional brain MRI 2 years after completing a physical activity treatment. *The journals of gerontology. Series A, Biological sciences and medical sciences.* 2010;65(6):639<647.

48. Ruscheweyh R, Willemer C, Kruger K, et al. Physical activity and memory functions: an interventional study. *Neurobiology of aging.* 2011;32(7):1304<1319.

49. Voss MW, Heo S, Prakash RS, et al. The influence of aerobic fitness on cerebral white matter integrity and cognitive function in older adults: results of a one<year exercise intervention. *Human brain mapping.* 2013;34(11):2972<2985.

50. Voss MW, Erickson KI, Prakash RS, et al. Neurobiological markers of exercise<related brain plasticity in older adults. *Brain, behavior, and immunity.* 2013; 28:90<99.

51. Predovan D, Fraser SA, Renaud M, Bherer L. The effect of three months of aerobic training on stroop performance in older adults. *Journal of aging research.* 2012; 2012:269815.

52. Baker LD, Frank LL, Foster<Schubert K, et al. Effects of aerobic exercise on mild cognitive impairment: a controlled trial. *Archives of neurology.* 2010;67(1):71<79.
