# Supplementary material for: Cardiorespiratory fitness is associated with cognitive function in late adulthood: baseline findings from the IGNITE study
Source: Br J Sports Med. 2024 Dec 10;59(3):e108257. doi: 10.1136/bjsports-2024-108257 (PMC11790366; doi:10.1136/bjsports-2024-108257)
Supplement: online supplemental file 2 [file bjsports-59-3-s002.pdf]

SUPPLEMENTAL MATERIAL

A.1. Cardiorespiratory Fitness Testing

Prior to each VO_2 test, the metabolic cart was calibrated with a 3-liter syringe at room temperature along with gas calibration with known levels of oxygen, carbon dioxide, and nitrogen. Manufacture recommended quality control was also performed every six months. The University of Pittsburgh and University of Kansas Medical Center used Parvo Medics TrueOne 2400; Northeastern University used COSMED Quark CPET.

A.2. Cognitive Assessment Measures

Participants completed a comprehensive neuropsychological evaluation administered by annually-certified psychometricians and delivered across two days. Each task is described below. All computerized cognitive tests were scripted in E-prime 3.0 and administered via a standard monitor and keyboard.

Montreal Cognitive Assessment (MoCA): The MoCA is a widely used and validated screening instrument for detecting cognitive impairment (Nasreddine et al., 2005). The MoCA involves brief assessments of short-term memory, visuospatial abilities, orientation, attention, language, and executive functions, which are scored and summed to calculate a total score. Participants can receive up to 30 points on the MoCA. To provide a more comprehensive assessment of Clock Drawing performance for the purposes of examining domain-specificity in the Confirmatory Factor Analysis (CFA), we applied the well-validated and widely used Rouleau scoring criteria

(Rouleau et al., 1992). Possible scores range from 0-10 based on integrity of the clock face, presence and sequencing of numbers, and presence and placement of clock hands.

Logical Memory: This subtest from the Wechsler Memory Scale (Wechsler, 1997) assesses contextual episodic memory. The computerized version adapted by Salthouse and colleagues as part of the Virginia Cognitive Aging Project (VCAP) was used. Participants were orally presented with two narrative stories, one presented once (Story A) and the other presented twice (Story B). They were asked to freely recall each story immediately after presentation and again after a 20-minute delay. The outcome variables were raw total immediate and delayed recall scores.

Paired Associates: A modified version from the Wechsler Memory Scale (Wechsler, 1997) adapted as part of VCAP (Salthouse & Ferrer-Caja, 2003; Salthouse et al., 1996) was used. Participants were orally presented with two separate word lists, each containing six word pairs (e.g., bell-pencil, bank-clown). Participants were then presented with the first word in each pair and instructed to freely recall the word associated with each stimulus word immediately and following a 20-minute delay. Immediate and delayed recall performance were measured as the mean number of words recalled across both lists. This study focused on mean immediate and delayed recall raw scores.

Hopkins Verbal Learning Test-Revised (HVLT-R): This test was designed to measure verbal episodic memory (Brandt, 1991, 2001). Participants were orally presented with a word list consisting of 12 items from three semantic categories (animals, stones, shelter), and were

instructed to recall the words over three learning trials, and once again after a 20-minute delay. Following delayed recall, participants were read 24 words and asked to identify whether each word was on the list (target) or not (distractor). A recognition discrimination index was calculated by subtracting the number of false positives from true positives. This study focused on total learning, delayed recall, and recognition discrimination index raw scores.

Picture Sequence Memory Test (PSMT): This NIH Toolbox test was designed to measure visual episodic memory (Zelazo et al., 2013). Using an iPad and an iPad keyboard, pictures of objects and activities were presented one at a time in a fixed, sequential order. Then, the pictures were displayed in a random spatial arrangement and participants were instructed to move each picture to its correct location to replicate the sequence presented. Raw scores reflect the cumulative number of adjacent pairs of pictures remembered correctly over the three trials.

Brief Visuospatial Memory Test-Revised (BVMT): This is a widely used measure of visual episodic memory (Benedict et al., 1996). Participants are shown a page containing six geometric figures in a 2x3 matrix. Participants were asked to immediately reproduce the designs in the correct location over three learning trials, and once again following a 25-minute delay. On the recognition trial, participants were shown 12 designs and asked determine whether each was part of the matrix (target) or not (foil). This study focused on immediate and delayed recall.

Trail Making Test: The Trail Making Test (TMT) is a paper-and-pencil measure comprised of two subtests (Reitan, 1958). TMT Part A is a measure of psychomotor processing speed that requires participants to connect numbers displayed on a page in ascending order. TMT Part B

measures set-shifting and requires participants to alternate between numbers and letters to connect them in ascending order. Time to completion (seconds) was measured, with lower values reflecting better performance. The signs of TMT Part A and Part B were inversed (e.g., higher scores reflect better cognitive performance) to ensure measurement consistency across variables entered into the CFA.

Stroop task: This task is a computerized measure of inhibitory control. Participants were asked to identify the ink color for congruent, neutral, and incongruent stimuli. On congruent items, the word matched the ink color in which the word was printed (e.g., RED in red ink). Neutral stimuli were matched for frequency and word length with the color words, but the meanings of the words were unrelated to the ink color (e.g., SHIP in red ink). On incongruent items, the printed word was conflicting with the ink color (e.g., RED in blue ink). Accuracy and response times were recorded, and Stroop incongruent response time was used as the primary outcome in this study. Stroop incongruent response time was inversed (e.g., higher scores reflect better cognitive performance) to ensure measurement consistency across variables entered into the CFA.

Flanker task: This NIH Toolbox task is delivered via iPad and measures attention and inhibitory control. Participants were shown a screen displaying an arrow flanked by two other arrows on each side, and were instructed to choose one of two buttons on the screen that corresponded to the direction in which the middle arrow is pointing. On congruent trials, all of the arrows were pointing in the same direction. On incongruent trials, the flanking arrows were pointing in the opposite direction of the middle arrow. Incongruent trials require the participant to focus on a particular stimulus while inhibiting the flanking stimuli (Zelazo et al., 2013). Congruent and incongruent

trials were mixed. The NIH Toolbox calculated a Flanker Inhibitory Control score based on a combination of accuracy and reaction time (RT) data (Zelazo et al., 2014).

Dimensional Change Card Sort (DCCS) test: This NIH Toolbox task is delivered via iPad and measures attention and cognitive flexibility. Two target pictures were presented that vary along two dimensions (e.g., shape and color). Participants were asked to match a series of bivalent test pictures (e.g., yellow balls and blue trucks) to the target pictures, first according to one dimension (e.g., color) and then, after a number of trials, according to the other dimension (e.g., shape). The relevant dimension for sorting was indicated by a cue word (e.g., "shape" or "color") that appeared on the screen. The NIH Toolbox calculated a DCCS score based on a combination of accuracy and RT data (Zelazo et al., 2014).

Digit Symbol Substitution Test (DSST): This test was designed to assess psychomotor processing speed and basic attention (WAIS-III)(Wechsler, 1997)). Participants were asked to match symbols to numbers according to a key as quickly as possible for 90 seconds. The raw score was calculated by summing the total number of correct responses.

Matrix reasoning: This computer-administered test assesses visuospatial processing and reasoning (Raven, 1962) and was adapted by Salthouse and colleagues as part of VCAP. Participants were shown a matrix with a missing piece, and were asked to determine which pattern from a set of options best fits the missing cell to complete the matrix. The primary outcome was the percentage of correct responses.

Spatial Relations: The Spatial Relations test is a computer-administered measure of visuoconstructional ability (Bennett et al., 1947) adapted by Salthouse and colleagues as part of VCAP. Participants were shown a two-dimensional pattern and were asked to determine which three-dimensional object matches the two-dimensional pattern. The primary outcome was the percentage of correct items.

*Letter Comparison test***:** This test originated from the VCAP and was designed to measure processing speed (Salthouse & Babcock, 1991). Participants were asked to determine whether two strings of letters were the same or different by writing "S" on the line between two letter strings if they were identical, or "D" if they differed. The test was stopped after 30 seconds and participants were asked to work as quickly and accurately as possible. Two separate trials were consecutively administered with each including 21 letter sets. The primary outcome was the average of the total correct responses of both trials.

N-Back Working Memory Task: This is a measure of visual working memory, which was completed by participants during their MRI scan. On the 1-back condition, participants were instructed to indicate whether the letter on the screen matched the letter that was previously displayed. The 2-back condition requires a greater cognitive load, and instructed participants to indicate whether the letter on the screen matched the letter that was displayed two trials previously. Blocks of the 1-back and 2-back conditions were interspersed by blocks of a visual cross-hair (Drake et al., 2022). Accuracy and reaction time for both conditions was recorded.

List Sorting Working Memory (LSWM) Test: This NIH Toolbox Test measures working memory and was delivered via iPAD (Tulsky et al., 2014; Weintraub et al., 2014). Participants were asked to recall and sequence by size different stimuli that were presented visually and via audio. If the participant sorted the list correctly, another longer list of familiar stimuli (foods and/or animals) was presented. On 1-list trials, all stimuli were from the same category (foods or animals). On 2-list trials, the stimuli were from two different categories (foods *and* animals). The participant was asked to sequence the food stimuli followed by the animal stimuli. The main outcome was total items correct across the 1-list and 2-list conditions (maximum 28).

Spatial Working Memory: This is a computerized measure of spatial working memory (Erickson et al., 2011). Participants were shown two, three, or four black dots at random locations on the screen. Then, the black dots were removed and a red dot appeared in either one of the same locations as the target dots (match condition) or at a different location (nonmatch condition). After each trial, the participant was asked to determine whether the red dot was in the same location or a different location from the black dots previously presented. The main outcome measures were accuracy and response times.

Antonym and Synonym test: This computerized VCAP test was used to assess verbal intelligence (Salthouse, 1993). In this task, participants were asked to identify from a total of 20 trials which of five-word choices is the correct synonym or antonym to a presented target word.

Verbal fluency: This test was designed to assess letter (phonemic) and category (semantic) fluency. The participant was given 60 seconds to produce words that begin with a given letter (Trial 1 = 'F', Trial 2 = 'A', and Trial 3 = 'S') (Newcombe, 1969), and those that belong to a given category ('Animal' = Trial 4) (Benton, 1968), while adhering to certain restrictions (e.g., no proper nouns or variations of the same word). Total correct words for letter fluency and category fluency were used as outcomes.

Iowa Gambling Test: On this computerized measure of decision-making (Bechara et al., 1994) participants were asked to maximize fictional 'money' from an initial pool by selecting cards from 4 decks. The decks have varying risk-reward ratios, such that two decks dispense relatively lower yet stable profits, while the other two dispense higher but unpredictable profits/losses (with an ultimately lower yield).

Task-switching test: On this computerized measure of set-shifting, participants had to indicate (I) whether the number presented in a 'circle' was lower or higher than 5, or (II) whether the number presented in a 'square' was odd or even, by pressing two buttons on the keyboard. After practicing these two tasks, the participant completed a block of circle-only trials, then a block of square-only trials, and finally the circle and square trials were intermixed. Response times and accuracy rates were recorded.

Cohen's Relational Memory Test: This is a spatial reconstruction task developed by Cohen and colleagues (Monti et al., 2015). Participants studied the arrangement of five separate line drawings on a computer screen for 10 seconds, after which the objects disappeared. Following a brief delay, the stimuli reappeared, aligned at the top of the screen, and participants were asked to move each stimulus to the location it was originally positioned. There were several practice trials followed by 15 test trials. We focused on four scores: misplacement, edge resizing, distortion, and swaps (Monti et al., 2015; Watson et al., 2013)

A.3. Statistical Analyses

Dimensionality reduction of the cognitive data

Confirmatory factor analysis (CFA) was used to determine the latent cognitive architecture of the cognitive tasks. The *a priori* five-factor model and the five alternative models are specified in Table S6. The initial hypothesized model (model 1) was compared to another five-factor model with a different set of factors (model 2), two different second-order five-factor models (model 3 and 4), a four-factor model (model 5), and a second-order four-factor model (model 6).

Seven different goodness-of-fit statistics were used to assess model fit, including the χ^2, the χ^2/df ratio, the comparative fit index (CFI), the Tucker-Lewis Index (TLI), the root mean square error of approximation (RMSEA), the Standardized Root Mean Square Residual (SRMR), and the Akaike Information Criterion (AIC). A model that has a χ^2/df ratio of < 3 was considered to fit the observed data well (Carmines & McIver, 1981). A CFI and TLI ≥ 0.90 was an indication of a good fit, while values > 0.95 indicated a close fit (Hu & Bentler, 1999; Schretlen et al., 2013). RMSEA and SRMR values < 0.05 indicated a close fit, while values < 0.08

indicated a reasonable error of approximation (Browne & Cudeck, 1992; Byrne, 2012). The AIC compared models and lower values indicated a better fit (Akaike, 1974).

Based on model 1, in model 2, we dropped the language factor, slightly adjusted the set of tests, and divided the executive function factor into working memory and attentional control. In the second-order five-factor models (model 3 and model 4), we created a second-order latent factor representing general cognitive ability. In the four-factor model (model 5), we grouped measures included in the working memory and attentional control factors together as a general executive function factor and left the other dimensions in model 2 unchanged. Finally, we defined a second-order four-factor model (model 6), with attentional control and working memory as two sub-factors nested under a higher-order executive function factor.

Testing associations between CRF components

We examined associations between VO_{2max} and American College of Sports Medicine (ACSM) criteria for determining maximal CRF (Liguori, 2020). We examined the relationship between VO_{2max} and continuous measures of the 1) maximum Respiratory Exchange Ratio (RER), 2) maximum Rating of Perceived Exertion (RPE), and 3) maximum heart rate using Pearson correlations. We used a point-biserial correlation to assess the relationship between VO_{2max} and plateau in VO_2 between two or more workloads (increase less than 0.15 L/min or 2.0 ml/kg/min during the last minute of corresponding workloads).

B. Supplemental Results

CFA Results. Table S7 shows the goodness-of-fit statistics for each hypothesized model. The five-factor model with language factor (model 1) provided a moderate description of the latent factor structure (χ^2/df = 2.900, CFI = 0.928, TLI = 0.918, RMSEA = 0.054, SRMR=0.061). The five-factor model with working memory and executive function/attentional control (model 2) represented a significant improvement over the initial model after dropping the language factor, and yielded more promising results in model fit (χ^2/df = 2.649, CFI = 0.945, TLI = 0.936, RMSEA = 0.05, SRMR = 0.05). Both second-order models (models 3 and 4) in which all latent factors were loaded to a global cognitive ability factor yielded poorer goodness-of-fit statistics. The four-factor model (model 5) in which we grouped the measures in working memory and attentional control as a general executive function factor yielded a better fit than model 1, but performed worse than model 2. The second-order four-factor model (model 6) showed a slight improvement over model 5 but did not outperform model 2. Overall, the five-factor model without language showed the best fit to the observed data in all goodness-of-fit statistics. The executive function factor is labeled as executive function/attentional control to reflect both the overarching domain and the specific executive processes encompassed within the factor.

C. Supplemental Tables

Table S1. *Results from moderation models examining conditional associations between VO_{2max} and cognitive performance as a function of age.*

Predictors	Episodic Memory				Processing Speed				Working Memory				EF/Attentional Control				Visuospatial			
	B	β	t	p	B	β	t	p	B	β	t	p	B	β	t	p	B	β	t	p
VO_{2max}	0.055	0.453	0.669	0.503	0.025	0.199	0.295	0.768	0.056	0.520	0.790	0.430	0.044	0.373	0.560	0.575	0.079	0.710	1.078	0.281
Age	-0.013	-0.080	-0.497	0.620	-0.042	-0.250	-1.564	0.118	-0.024	-0.164	-1.050	0.294	-0.035	-0.222	-1.410	0.159	-0.012	-0.079	-0.508	0.611
VO_{2max} x Age	-0.001	-0.305	-0.453	0.651	-0.0001	-0.052	-0.078	0.938	-0.0004	-0.285	-0.436	0.663	-0.0003	-0.175	-0.264	0.792	-0.001	-0.464	-0.708	0.479
Sex	-0.339	-0.249	-6.077	<.001	-0.151	-0.109	-2.673	0.008	-0.084	-0.070	-1.746	0.081	-0.050	-0.038	-0.952	0.341	-0.047	-0.038	-0.955	0.340
Education	0.073	0.260	6.846	<.001	0.066	0.233	6.152	<.001	0.070	0.285	7.697	<.001	0.068	0.251	6.722	<.001	0.081	0.318	8.591	<.001
BMI	0.005	0.042	0.939	0.348	-0.002	-0.020	-0.437	0.662	0.008	0.082	1.865	0.063	0.002	0.023	0.514	0.607	0.011	0.114	2.599	0.010
APOE4 carriage	-0.074	-0.054	-1.459	0.145	-0.043	-0.031	-0.846	0.398	-0.054	-0.044	-1.241	0.215	-0.042	-0.032	-0.877	0.381	-0.027	-0.021	-0.591	0.554
	Adjusted R^2 = 0.157, p < 0.001				Adjusted R^2 = 0.162, p < 0.001				Adjusted R^2 = 0.202 , p < 0.001				Adjusted R^2 = 0.184, p < 0.001				Adjusted R^2 = 0.202, p < 0.001			

Table S2. *Results from moderation models examining conditional associations between VO_{2max} and cognitive performance as a function of sex.*

Predictors	Episodic Memory				Processing Speed				Working Memory				EF/Attentional Control				Visuospatial			
	B	β	t	p	B	β	t	p	B	β	t	p	B	β	t	p	B	β	t	p
VO_{2max}	0.023	0.191	3.066	0.002	0.029	0.235	3.810	<.001	0.032	0.297	4.924	<.001	0.033	0.281	4.609	<.001	0.031	0.281	4.637	<.001
Sex	-0.074	-0.055	-0.319	0.750	0.404	0.293	1.723	0.085	0.260	0.217	1.303	0.193	0.443	0.337	2.007	0.045	0.151	0.122	0.729	0.466
VO_{2max} x Sex	-0.012	-0.216	-1.169	0.243	-0.024	-0.447	-2.435	0.015	-0.015	-0.318	-1.773	0.077	-0.022	-0.417	-2.302	0.022	-0.009	-0.177	-0.985	0.325
Age	-0.024	-0.148	-3.780	<.001	-0.043	-0.259	-6.638	<.001	-0.033	-0.227	-5.962	<.001	-0.041	-0.260	-6.748	<.001	-0.028	-0.185	-4.841	<.001
Education	0.071	0.257	6.753	<.001	0.064	0.227	6.025	<.001	0.069	0.280	7.579	<.001	0.066	0.246	6.592	<.001	0.080	0.314	8.491	<.001
BMI	0.006	0.051	1.124	0.262	0.000	0.001	0.026	0.979	0.009	0.096	2.152	0.032	0.004	0.042	0.930	0.353	0.012	0.121	2.708	0.007
APOE4 carriage	-0.071	-0.051	-1.396	0.163	-0.038	-0.027	-0.752	0.452	-0.050	-0.041	-1.157	0.248	-0.037	-0.028	-0.780	0.436	-0.024	-0.019	-0.524	0.601
	Adjusted R^2 = 0.159, p < 0.001				Adjusted R^2 = 0.170 , p < 0.001				Adjusted R^2 = 0.206, p < 0.001				Adjusted R^2 = 0.191, p < 0.001				Adjusted R^2 = 0.203 , p < 0.001			

Table S3. *Results from moderation models examining conditional associations between VO_{2max} and cognitive performance as a function of years of education.*

Predictors	Episodic Memory				Processing Speed				Working Memory				EF/Attentional Control				Visuospatial			
	B	β	t	p	B	β	t	p	B	β	t	p	B	β	t	p	B	β	t	p
VO_{2max}	0.075	0.615	2.101	0.036	0.103	0.829	2.845	0.005	0.080	0.746	2.621	0.009	0.094	0.799	2.776	0.006	0.072	0.641	2.247	0.025
Education	0.145	0.520	3.148	0.002	0.173	0.613	3.734	<.001	0.140	0.569	3.546	<.001	0.158	0.585	3.610	<.001	0.137	0.536	3.337	<.001
VO_{2max} x Education	-0.003	-0.591	-1.621	0.105	-0.005	-0.863	-2.378	0.018	-0.003	-0.648	-1.827	0.068	-0.004	-0.759	-2.120	0.034	-0.003	-0.500	-1.407	0.160
Age	-0.024	-0.146	-3.718	<.001	-0.043	-0.256	-6.562	<.001	-0.033	-0.225	-5.902	<.001	-0.041	-0.257	-6.681	<.001	-0.027	-0.183	-4.785	<.001
Sex	-0.338	-0.248	-6.073	<.001	-0.150	-0.108	-2.666	0.008	-0.083	-0.069	-1.733	0.084	-0.050	-0.038	-0.937	0.349	-0.047	-0.037	-0.940	0.347
BMI	0.005	0.047	1.041	0.298	-0.001	-0.012	-0.260	0.795	0.008	0.087	1.985	0.048	0.003	0.029	0.664	0.507	0.012	0.117	2.670	0.008
APOE4 carriage	-0.068	-0.049	-1.346	0.179	-0.036	-0.026	-0.705	0.481	-0.049	-0.040	-1.117	0.265	-0.036	-0.027	-0.743	0.458	-0.022	-0.017	-0.479	0.632
	Adjusted R^2 = 0.160, p < 0.001				Adjusted R^2 = 0.170 , p < 0.001				Adjusted R^2 = 0.206 , p < 0.001				Adjusted R^2 = 0.190, p < 0.001				Adjusted R^2 = 0.204 , p < 0.001			

Table S4. *Results from moderation models examining conditional associations between VO_{2max} and cognitive performance as a function of APOE4 carrier status.*

Predictors	Episodic Memory				Processing Speed				Working Memory				EF/Attentional Control				Visuospatial			
	B	β	t	p	B	β	t	p	B	β	t	p	B	β	t	p	B	β	t	p
VO_{2max}	0.018	0.146	2.583	0.010	0.015	0.123	2.179	0.030	0.026	0.243	4.428	<.001	0.022	0.191	3.432	0.001	0.031	0.275	5.008	<.001
APOE4 carriage	-0.086	-0.062	-0.404	0.686	-0.234	-0.167	-1.086	0.278	0.012	0.010	0.066	0.947	-0.097	-0.073	-0.479	0.632	0.191	0.151	1.005	0.315
VO_{2max} x APOE	0.001	0.010	0.063	0.950	0.009	0.141	0.911	0.363	-0.003	-0.056	-0.368	0.713	0.003	0.043	0.282	0.778	-0.010	-0.177	-1.173	0.241
Age	-0.025	-0.150	-3.816	<.001	-0.043	-0.261	-6.680	<.001	-0.033	-0.230	-6.017	<.001	-0.042	-0.262	-6.796	<.001	-0.028	-0.187	-4.900	<.001
Sex	-0.338	-0.249	-6.070	<.001	-0.149	-0.108	-2.652	0.008	-0.084	-0.070	-1.751	0.080	-0.050	-0.038	-0.943	0.346	-0.049	-0.039	-0.978	0.328
Education	0.072	0.259	6.823	<.001	0.066	0.232	6.123	<.001	0.070	0.284	7.693	<.001	0.068	0.250	6.703	<.001	0.081	0.318	8.609	<.001
BMI	0.004	0.041	0.913	0.362	-0.002	-0.022	-0.495	0.621	0.008	0.082	1.864	0.063	0.002	0.021	0.484	0.628	0.011	0.115	2.634	0.009
	Adjusted R^2 = 0.157, p < 0.001				Adjusted R^2 = 0.163 , p < 0.001				Adjusted R^2 = 0.202, p < 0.001				Adjusted R^2 = 0.184, p < 0.001				Adjusted R^2 = 0.203 , p < 0.001			

Table S5. *Results from moderation models examining conditional associations between VO_{2max} and cognitive performance as a function of beta-blocker medication use.*

Predictors	Episodic Memory				Processing Speed				Working Memory				EF/Attentional Control				Visuospatial			
	B	β	t	p	B	β	t	p	B	β	t	p	B	β	t	p	B	β	t	p
VO_{2max}	0.015	0.125	2.347	0.019	0.012	0.101	1.910	0.057	0.023	0.213	4.106	<.001	0.019	0.162	3.107	0.002	0.026	0.231	4.458	<.001
Beta-blocker	-0.274	-0.160	-1.007	0.314	-0.612	-0.352	-2.234	0.026	-0.308	-0.203	-1.319	0.188	-0.534	-0.322	-2.068	0.039	-0.251	-0.160	-1.034	0.302
VO_{2max} x Beta-blocker	0.010	0.116	0.745	0.457	0.025	0.288	1.856	0.064	0.015	0.201	1.324	0.186	0.025	0.305	1.989	0.047	0.014	0.175	1.150	0.251
Age	-0.025	-0.153	-3.891	<.001	-0.045	-0.269	-6.889	<.001	-0.034	-0.234	-6.108	<.001	-0.043	-0.270	-6.974	<.001	-0.028	-0.190	-4.949	<.001
Sex	-0.331	-0.244	-5.912	<.001	-0.140	-0.101	-2.477	0.014	-0.083	-0.069	-1.726	0.085	-0.048	-0.036	-0.896	0.370	-0.050	-0.040	-0.991	0.322
Education	0.072	0.257	6.755	<.001	0.065	0.230	6.089	<.001	0.070	0.284	7.689	<.001	0.068	0.251	6.724	<.001	0.081	0.318	8.592	<.001
BMI	0.004	0.041	0.912	0.362	-0.002	-0.021	-0.463	0.644	0.008	0.080	1.829	0.068	0.002	0.021	0.478	0.633	0.011	0.112	2.551	0.011
APOE4 carriage	-0.074	-0.054	-1.464	0.144	-0.045	-0.032	-0.876	0.382	-0.053	-0.043	-1.219	0.223	-0.042	-0.031	-0.869	0.385	-0.025	-0.020	-0.547	0.584
	Adjusted R^2 = 0.158, $p < 0.001$				Adjusted R^2 = 0.170 , $p < 0.001$				Adjusted R^2 = 0.203, $p < 0.001$				Adjusted R^2 = 0.189, $p < 0.001$				Adjusted R^2 = 0.202 , $p < 0.001$			

For all moderation analyses, we employed multiple linear regression models, including CRF (VO_{2max}), the moderator variable, and their interaction product, controlling for demographics. *APOE4* carriage = non-carrier: 0, carrier: 1; Sex = male:1, female: 0. Site was also included as a covariate in all models (data not shown). BMI = body mass index; EF = executive function.

Table S6. *Model specifications for confirmatory factor analysis.*

Model	Factors	Measures
Model 1: Five-factor model *(w/ Language)*	Episodic Memory	Logical memory, HVLT, paired associates, picture sequence, BVMT, MoCA-delayed recall
	Processing Speed	DSST, letter comparison, TMT-A
	Language	Letter fluency, VCAP Synonym/Antonym
	Executive Function	Spatial working memory, N-back, list sort, TMT-B, DCCS, Stroop, Flanker
	Visuospatial	Spatial relations, MoCA-clock draw, matrix reasoning
Model 2: Five-factor model *(w/o Language, w/ AC&WM)*	Episodic Memory	Logical memory, HVLT, paired associates, picture sequence, BVMT, MoCA-delayed recall
	Processing Speed	DSST, letter comparison, TMT-A
	Working Memory	Spatial working memory, N-back, list sort
	Attentional Control	TMT-B, DCCS, Stroop, Flanker
	Visuospatial	Spatial relations, MoCA-clock draw, matrix reasoning
Model 3: Second-order five-factor model *(w/ Language)*	Cognitive Ability (second-order)	Episodic Memory, Processing Speed, Language, Executive Function, Visuospatial
	Episodic Memory (first-order)	Logical memory, HVLT, paired associates, picture sequence, BVMT, MoCA-delayed recall
	Processing Speed (first-order)	DSST, letter comparison, TMT-A
	Language (first-order)	Letter fluency, VCAP Synonym/Antonym
	Executive Function (first-order)	Spatial working memory, N-back, list sort, TMT-B, DCCS, Stroop, Flanker
	Visuospatial (first-order)	Spatial relations, MoCA-clock draw, matrix reasoning
Model 4: Second-order five-factor model *(w/o Language, w/ AC&WM)*	Cognitive Ability (second-order)	Episodic Memory, Processing Speed, Working Memory, Attentional Control, Visuospatial
	Episodic Memory (first-order)	Logical memory, HVLT, paired associates, picture sequence, BVMT, MoCA-delayed recall
	Processing Speed (first-order)	DSST, letter comparison, TMT-A
	Working Memory (first-order)	Spatial working memory, N-back, list sort
	Attentional Control (first-order)	TMT-B, DCCS, Stroop, Flanker
	Visuospatial (first-order)	Spatial relations, MoCA-clock draw, matrix reasoning
Model 5: Four-factor model *(w/ EF)*	Episodic Memory	Logical memory, HVLT, paired associates, picture sequence, BVMT, MoCA-delayed recall
	Processing Speed	DSST, letter comparison, TMT-A
	Executive Function	Spatial working memory, N-back, list sort, TMT-B, DCCS, Stroop, Flanker
	Visuospatial	Spatial relations, MoCA-clock draw, matrix reasoning
Model 6: Second-order four-factor model *(AC&WM nested under EF)*	Episodic Memory	Logical memory, HVLT, paired associates, picture sequence, BVMT, MoCA-delayed recall
	Processing Speed	DSST, letter comparison, TMT-A
	Visuospatial	Spatial relations, MoCA-clock draw, matrix reasoning
	Executive Function (second-order)	Working Memory, Attentional Control
	Working Memory (first-order)	Spatial working memory, N-back, list sort
	Attentional Control (first-order)	TMT-B, DCCS, Stroop, Flanker

Table S7. *Goodness-of-fit statistics for confirmatory factor analyses*

Model	χ^2	df	χ^2/df	RMSEA	CFI	TLI	SRMR	AIC
Model 1: Five-factor model (w/ Language)	968.766	334	2.900	0.054	0.928	0.918	0.061	43491.23
Model 2: Five-factor model (w/o Language, w/ AC&WM)	685.991	259	2.649	0.05	0.945	0.936	0.05	38800.08
Model 3: Second-order five-factor model (w/ Language)	1097.723	339	3.238	0.059	0.914	0.904	0.068	43610.19
Model 4: Second-order five-factor model (w/o Language, w/ AC&WM)	774.715	264	2.935	0.055	0.934	0.925	0.063	38878.80
Model 5: Four-factor model (w/ EF)	758.348	263	2.883	0.054	0.936	0.927	0.059	38814.43
Model 6: Second-order four-factor model (EF on top of AC&WM)	728.371	261	2.791	0.053	0.940	0.931	0.058	38788.46

D. Supplemental Figures

Figure S1. Associations between age, education and the cognitive composites.

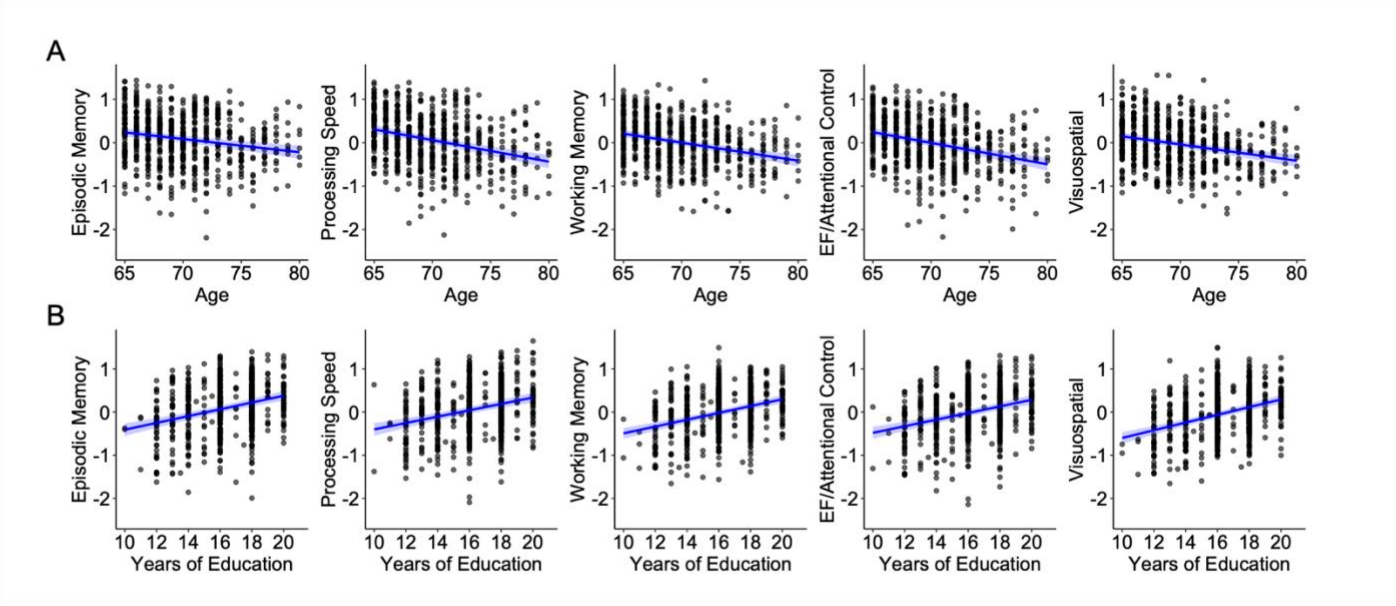

A) Scatterplots of the relationship between age and the five cognitive composites adjusted for sex, site, education, and body mass index (BMI). B) Scatterplots of the relationship between years of education and the five cognitive composites adjusted for age, sex, site, and BMI. EF = executive function.

Works Cited:

Akaike, H. (1974). A New Look at the Statistical Model Identification. *IEEE Transactions on Automatic Control*, *19*(6), 716-723. https://doi.org/10.1109/TAC.1974.1100705

Bechara, A., Damasio, A. R., Damasio, H., & Anderson, S. W. (1994). Insensitivity to future consequences following damage to human prefrontal cortex. *Cognition*, 50-50.

Benedict, R. H. B., Groninger, L., Schretlen, D., Dobraski, M., & Shpritz, B. (1996). Revision of the brief visuospatial memory test: Studies of normal performance, reliability, and, validity. *Psychological Assessment*, *8*(2), 145-153. https://doi.org/10.1037/1040-3590.8.2.145

Bennett, G. K., Seashore, H. G., & Wesman, A. G. (1947). *Differential aptitude tests*. Psychological Corporation.

Benton, A. L. (1968). Differential behavioral effects in frontal lobe disease. *Neuropsychologia*, *6*(1), 53-60. https://doi.org/10.1016/0028-3932(68)90038-9

Brandt, J. (1991). The Hopkins Verbal Learning Test: Development of a new memory test with six equivalent forms. *The Clinical Neuropsychologist*, *5*(2), 125-142. https://doi.org/10.1080/13854049108403297

Brandt, J. (2001). Hopkins verbal learning test. *Clinical Neuropsychologist*. https://psycnet.apa.org/getdoi.cfm?doi=10.1037/t49859-000

Browne, M., & Cudeck, R. (1992). Alternative Ways of Assessing Model Fit. *Sociological Methods & Research*, *21*(2).

Byrne, B. (2012). *Structural Equation Modeling with Mplus Basic Concepts, Applications, and Programming*. Routledge Taylor & Francis Group.

Carmines, E. G., & McIver, J. P. (1981). *Analyzing Models with Unobserved Variables*. Sage Publications, Inc.

Drake, J. A., Jakicic, J. M., Rogers, R. J., Aghjayan, S. L., Stillman, C. M., Donofry, S. D., Roecklein, K. A., Lang, W., & Erickson, K. I. (2022). Reduced brain activity during a working memory task in middle-aged apolipoprotein E ε4 carriers with overweight/obesity. *Frontiers in Human Neuroscience*, *16*, 1001229-1001229. https://doi.org/10.3389/FNHUM.2022.1001229/BIBTEX

Erickson, K. I., Voss, M. W., Prakash, R. S., Basak, C., Szabo, A., Chaddock, L., Kim, J. S., Heo, S., Alves, H., White, S. M., Wojcicki, T. R., Mailey, E., Vieira, V. J., Martin, S. A., Pence, B. D., Woods, J. A., McAuley, E., & Kramer, A. F. (2011). Exercise training increases size of hippocampus and improves memory. *Proceedings of the National Academy of Sciences of the United States of America*, *108*(7), 3017-3022. https://doi.org/10.1073/PNAS.1015950108/SUPPL_FILE/PNAS.201015950SI.PDF

Hu, L. T., & Bentler, P. M. (1999). Cutoff criteria for fit indexes in covariance structure analysis: Conventional criteria versus new alternatives. *Structural Equation Modeling: A Multidisciplinary Journal*, *6*(1), 1-55. https://doi.org/10.1080/10705519909540118

Liguori, G., & American College of Sports Medicine. (2020). *ACSM's guidelines for exercise testing and prescription.* Lippincott Williams & Wilkins.

Monti, J. M., Cooke, G. E., Watson, P. D., Voss, M. W., Kramer, A. F., & Cohen, N. J. (2015). Relating Hippocampus to Relational Memory Processing across Domains and Delays.

Journal of cognitive neuroscience, *27*(2), 234-234. https://doi.org/10.1162/JOCN_A_00717

Nasreddine, Z. S., Phillips, N. A., Bédirian, V., Charbonneau, S., Whitehead, V., Collin, I., Cummings, J. L., & Chertkow, H. (2005). The Montreal Cognitive Assessment, MoCA: A Brief Screening Tool For Mild Cognitive Impairment. *Journal of the American Geriatrics Society*, *53*(4), 695-699. https://doi.org/10.1111/J.1532-5415.2005.53221.X

Newcombe, F. (1969). *Missile wounds of the brain: A study of psychological deficits*. Oxford University Press. https://pure.mpg.de/pubman/faces/ViewItemOverviewPage.jsp?itemId=item_2366873

Raven, J. C. (1962). *Advanced progressive matrices: Sets I and II*.

Reitan, R. M. (1958). Validity of the Trail Making Test as an Indicator of Organic Brain Damage. *Perceptual and Motor Skills*, *8*(3), 271-276. https://doi.org/10.2466/PMS.1958.8.3.271

Rouleau, I., Salmon, D. P., Butters, N., Kennedy, C., & McGuire, K. (1992). Quantitative and qualitative analyses of clock drawings in Alzheimer's and Huntington's disease. *Brain Cogn*, *18*(1), 70-87. https://doi.org/10.1016/0278-2626(92)90112-y

Salthouse, T. A. (1993). Speed and Knowledge as Determinants of Adult Age Differences in Verbal Tasks. *Journal of Gerotology*, *48*(1), 29-36.

Salthouse, T. A., & Babcock, R. L. (1991). Developmental Psychology Decomposing Adult Age Differences in Working Memory. *27*(5), 763-776.

Salthouse, T. A., & Ferrer-Caja, E. (2003). What Needs to Be Explained to Account for Age-Related Effects on Multiple Cognitive Variables? https://doi.org/10.1037/0882-7974.18.1.91

Salthouse, T. A., Fristoe, N., & Rhee, S. H. (1996). How Localized Are Age-Related Effects on Neuropsychological Measures? *Neuropsychology Psychological Association, Inc*, *0*(2), 272-285.

Schretlen, D. J., Pena, J., Aretouli, E., Orue, I., Cascella, N. G., Pearlson, G. D., & Ojeda, N. (2013). Confirmatory factor analysis reveals a latent cognitive structure common to bipolar disorder, schizophrenia, and normal controls. *Bipolar Disord*, *15*(4), 422-433. https://doi.org/10.1111/bdi.12075

Tulsky, D. S., Carlozzi, N., Chiaravalloti, N. D., Beaumont, J. L., Kisala, P. A., Mungas, D., Conway, K., & Gershon, R. (2014). NIH Toolbox Cognition Battery (NIHTB-CB): List Sorting Test to Measure Working Memory. *Journal of the International Neuropsychological Society*, *20*(6), 599-610. https://doi.org/10.1017/S135561771400040X

Watson, P. D., Voss, J. L., Warren, D. E., Tranel, D., & Cohen, N. J. (2013). Spatial reconstruction by patients with hippocampal damage is dominated by relational memory errors. *Hippocampus*, *23*(7), 570-580. https://doi.org/10.1002/HIPO.22115

Wechsler, D. (1997). *Wechsler Memory Scale* (Third ed.). The Psychological Corporation.

Weintraub, S., Dikmen, S. S., Heaton, R. K., Tulsky, D. S., Zelazo, P. D., Slotkin, J., Carlozzi, N. E., Bauer, P. J., Wallner-Allen, K., Fox, N., Havlik, R., Beaumont, J. L., Mungas, D., Manly, J. J., Moy, C., Conway, K., Edwards, E., Nowinski, C. J., & Gershon, R. (2014). The Cognition Battery of the NIH Toolbox for Assessment of Neurological and Behavioral Function: Validation in an Adult Sample. *Journal of the International Neuropsychological Society : JINS*, *20*(6), 567-567. https://doi.org/10.1017/S1355617714000320

Zelazo, P. D., Anderson, J. E., Richler, J., Wallner-Allen, K., Beaumont, J. L., Conway, K. P., Gershon, R., & Weintraub, S. (2014). NIH Toolbox Cognition Battery (CB): Validation of Executive Function Measures in Adults. *Journal of the International Neuropsychological Society : JINS*, *20*(6), 620-620. https://doi.org/10.1017/S1355617714000472

Zelazo, P. D., Anderson, J. E., Richler, J., Wallner-Allen, K., Beaumont, J. L., & Weintraub, S. (2013). II. NIH TOOLBOX COGNITION BATTERY (CB): MEASURING EXECUTIVE FUNCTION AND ATTENTION. *Monographs of the Society for Research in Child Development*, *78*(4), 16-33. https://doi.org/10.1111/MONO.12032
